# *TP53*-Status-Dependent Oncogenic EZH2 Activity in Pancreatic Cancer

**DOI:** 10.3390/cancers14143451

**Published:** 2022-07-15

**Authors:** Lennart Versemann, Shilpa Patil, Benjamin Steuber, Zhe Zhang, Waltraut Kopp, Hannah Elisa Krawczyk, Silke Kaulfuß, Bernd Wollnik, Philipp Ströbel, Albrecht Neesse, Shiv K. Singh, Volker Ellenrieder, Elisabeth Hessmann

**Affiliations:** 1 Department of Gastroenterology, Gastrointestinal Oncology and Endocrinology, University Medical Center Goettingen, 37075 Goettingen, Germany; lennart.versemann@t-online.de (L.V.); shilpapatil528@gmail.com (S.P.); benjamin.steuber@med.uni-goettingen.de (B.S.); zhe.zhang@med.uni-goettingen.de (Z.Z.); wkopp@med.uni-goettingen.de (W.K.); albrecht.neesse@med.uni-goettingen.de (A.N.); shiv.singh@med.uni-goettingen.de (S.K.S.); volker.ellenrieder@med.uni-goettingen.de (V.E.); 2 Clinical Research Unit 5002, KFO5002, University Medical Center Göttingen, 37075 Göttingen, Germany; elisa.krawczyk@outlook.de (H.E.K.); silke.kaulfuss@med.uni-goettingen.de (S.K.); bernd.wollnik@med.uni-goettingen.de (B.W.); philipp.stroebel@med.uni-goettingen.de (P.S.); 3 Institute of Human Genetics, University Medical Center Goettingen, 37075 Goettingen, Germany; 4 Institute of Pathology, University Medical Center Goettingen, 37075 Goettingen, Germany

**Keywords:** *CDKN2A*, EZH2, epigenetics, p53, pancreatic cancer

## Abstract

**Simple Summary:**

Epigenetic alterations contribute to the aggressiveness and therapy resistance of Pancreatic Ductal Adenocarcinoma (PDAC). However, epigenetic regulators, including Enhancer of Zeste Homolog 2 (EZH2), reveal a strong context-dependent activity. Our study aimed to examine the context-defining molecular prerequisites of oncogenic EZH2 activity in PDAC to assess the therapeutic efficacy of targeting EZH2. Our preclinical study using diverse PDAC models demonstrates that the *TP53* status determines oncogenic EZH2 activity. Only in *TP53*-wildtype (wt) PDAC subtypes was EZH2 blockade associated with a favorable PDAC prognosis mainly through cell-death response. We revealed that EZH2 depletion increases p53wt stability by the de-repression of *CDKN2A*. Therefore, our study provides preclinical evidence that an intact *CDKN2A*-p53wt axis is indispensable for a beneficial outcome of EZH2 depletion and highlights the significance of molecular stratification to improve epigenetic targeting in PDAC.

**Abstract:**

Pancreatic Ductal Adenocarcinoma (PDAC) represents a lethal malignancy with a consistently poor outcome. Besides mutations in PDAC driver genes, the aggressive tumor biology of the disease and its remarkable therapy resistance are predominantly installed by potentially reversible epigenetic dysregulation. However, epigenetic regulators act in a context-dependent manner with opposing implication on tumor progression, thus critically determining the therapeutic efficacy of epigenetic targeting. Herein, we aimed at exploring the molecular prerequisites and underlying mechanisms of oncogenic Enhancer of Zeste Homolog 2 (EZH2) activity in PDAC progression. Preclinical studies in EZH2 proficient and deficient transgenic and orthotopic in vivo PDAC models and transcriptome analysis identified the *TP53* status as a pivotal context-defining molecular cue determining oncogenic EZH2 activity in PDAC. Importantly, the induction of pro-apoptotic gene signatures and processes as well as a favorable PDAC prognosis upon EZH2 depletion were restricted to p53 wildtype (wt) PDAC subtypes. Mechanistically, we illustrate that EZH2 blockade de-represses *CDKN2A* transcription for the subsequent posttranslational stabilization of p53wt expression and function. Together, our findings suggest an intact *CDKN2A*-p53wt axis as a prerequisite for the anti-tumorigenic consequences of EZH2 depletion and emphasize the significance of molecular stratification for the successful implementation of epigenetic targeting in PDAC.

## 1. Introduction

Polycomb group (PcG) proteins constitute a family of epigenetic regulators which install and maintain gene silencing [1]. PcG-dependent gene regulation and chromatin organization are heavily involved in developmental processes, in which the epigenetic regulators repress differentiation-associated gene signatures in a spatially and temporally-restricted manner, thus maintaining stem cell traits [2,3]. Consistent with their critical involvement in orchestrating cell fate transitions [4], PcG proteins play a pivotal role in cancer development and progression [5,6,7]. This is particularly true for the catalytic domain of the Polycomb Repressive Complex 2 (PRC2), the histone methyltransferase Enhancer of Zeste Homolog 2 (EZH2). In the canonical PRC2 pathway, EZH2 targets lysine 27 on histone 3 for trimethylation (H3K27me3), thus inducing transcriptional repression [8]. In many solid malignancies, EZH2 is found to be overexpressed [1], thus promoting oncogenic hallmarks like proliferation, invasion, and metastasis [9,10,11]. In line with these reports, we and others have linked abundant EZH2 expression and activity with the development and progression of pancreatic ductal adenocarcinoma (PDAC) [8,12,13,14,15]. 

Despite substantial scientific and clinical efforts, PDAC remains one of the most aggressive tumor entities with a 5-year survival rate of less than 10% [16]. Besides the signature mutations in the *KRAS*, *TP53*, *CDKN2A*, and *DPC4* genes and additional genetic events which occur at a lower frequency [17], the epigenomic landscape in general and chromatin alterations in particular significantly shape the different phenotypic states throughout pancreatic carcinogenesis and PDAC progression [8,13,18,19,20,21,22]. However, the expression of epigenetic regulators and their impact on transcriptional processes in the pancreas are dynamic and are critically determined by environmental or cell-intrinsic hierarchical signaling cues [22,23,24]. Consequently, diverse cellular and molecular contexts eventually translate into the antithetical functional consequences of epigenetic regulatory protein activity. This phenomenon has also been observed for EZH2-related functions in the pancreas. While EZH2 fosters acinar cell re-differentiation upon acinar cell damage and counteracts the formation of early pre-neoplastic pancreatic lesions upon PDAC initiation, aberrant EZH2 activity in advanced stages of pancreatic carcinogenesis promotes precursor cell proliferation and progression towards invasive PDAC [8,13,14]. In established PDAC, EZH2 fosters PDAC dedifferentiation and metastasis and acts as a transcriptional repressor of the *GATA6* gene, thus pushing PDAC cells into a more aggressive and chemo-resistant basal-like subtype state [13]. These findings suggest pharmacological interference with EZH2 activity or expression as a promising strategy to combat PDAC. Indeed, EZH2 inhibitors, such as Tazemetostat, have lately been approved for the treatment of epitheloid sarcoma [25] and are explored with regard to their anti-tumorigenic effect in advanced clinical trials in several malignancies (NCT03348631, NCT04204941, NCT04224493).

The previous evidence for the context-dependency of oncogenic EZH2 activity in the pancreas on one hand and the availability of EZH2-specific inhibitors on the other prompted us to explore the molecular conditions predicting a beneficial effect of targeting EZH2 in PDAC treatment. Our findings suggest that the anti-tumorigenic consequences of EZH2 depletion are p53-status-dependent and provide mechanistic evidence for an intact *CDKN2A*-p53 wildtype axis as a molecular prerequisite for implementing EZH2 inhibition as an effective therapeutic strategy in PDAC treatment. 

## 2. Material and Methods

### 2.1. Mouse Strains and In Vivo Experiments

Kras^G12D^ (KC), Kras^G12D^;Ezh^fl/fl^ (KEC), Kras^G12D^;Trp53^R172H/+^ (KPC), caNFATc1;Kras^G12D^ (NKC), Kras^G12D^;caNFATc1;Trp53^R172H/+^ (KNPC), and Kras^G12D^;caNFATc1;Trp53^fl/fl^ (KNP^null^C) mice have been described previously [13,26,27,28,29,30]. The genotyping of all mice was performed as previously described [29]. The extraction of primary PDAC cells derived from transgenic mice has been described previously [13,28,29,30]. The procedure for the generation of the orthotopic Panc-1 in vivo model has been conducted as stated before [13]. For the syngeneic model, 2 × 10^5^ Cas9 Ctrl and Cas9 *Ezh2* KO KPC cells were mixed with 50% matrigel and injected into the pancreatic tail of *C57BL/6J* mice. Sequences of the sgRNA and of the primers for knockout validation are depicted in Supplementary Table S1. Ezh2 knockout was verified prior to transplantation [13]. Small animal ultrasound was performed on all mice to evaluate tumor onset and progression. Mice were observed regarding general health symptoms and sacrificed when reaching endpoint criteria. All animal procedures were accomplished in agreement with the protocols approved by the Institutional Animal Care and Use Committee of the University Medical Center Goettingen (33.9-42502-04-14/1633,-15/2057,-19/3085,-17-2407).

### 2.2. Primary PDAC Tissue, Primary PDAC Cells, and Gene-Panel-Sequencing

Primary Patient-Derived PDAC cells (GöCDX5 and GöCDX13) were isolated from PDAC Patient-Derived-Xenograft (PDX) models as indicated before [13]. Briefly, PDX tumors with stable growth kinetics were subjected to harvesting and tissue dissociation utilizing the gentleMACS dissociator (Miltenyi Biotec, Bergisch Gladbach, Germany) combined with enzymatic dissociation with help of a human tumor dissociation kit (Miltenyi Biotec, Bergisch Gladbach, Germany). Upon dissociation, human tumor cells were positively selected utilizing a mouse cell depletion kit as per the manufacturers’ instructions (Miltenyi Biotec, Bergisch Gladbach, Germany) and were cultured on collagen type I coated dishes (Merck Millipore, Darmstadt, Germany). After 5 to 6 passages on collagen-coated plates, cells were transferred to uncoated plates for further expansion and experimental approaches. For DNA isolation from CDX cells, the DNeasy Blood & Tissue Kit (Quiagen, Hilden, Germany) was utilized according to manufacturer’s manual. For analysis in human primary PDAC, tumor samples were obtained from the Institute of Pathology at the University Medical Center Goettingen (UMG). Samples from resected PDAC specimens were either Formalin-Fixed Paraffin-Embedded (FFPE), as described previously [13], or were subjected to DNA isolation using DNeasy Blood & Tissue Kit (Quiagen, Hilden, Germany) following the manufacturer’s instructions. The molecular characterization of primary PDAC tissue and CDX cells was conducted in the Institute of Human Genetics (UMG) using gene panel sequencing. Briefly, targeted multigene panel sequencing was performed on 200 ng genomic DNA isolated from tumor biopsies. For library preparation, SureSelect^TM^ XTHS and QXT target enrichment Kit (Agilent Technologies, Santa Clara, CA, USA) with enzymatic fragmentation used following the manufacturer’s protocol (Agilent Technologies, Santa Clara, CA, USA). Libraries were sequenced on an Illumina NextSeq 550 with 2.5 High output chemistry and 150 bp read length. Sequence Pilot (jsi medical systems GmbH, Ettenheim, Germany) Software was used to align sequences to a human reference sequence (hg19) and for variant calling. Samples were screened for variants in *TP53* (ENST00000269305) and *CDKN2A* (ENST00000304494). Variants were assessed according to the American College of Medical Genetics (ACMG) guidelines [31] to identify likely pathogenic or pathogenic variants. The generation of translational PDAC models and their molecular characterization were approved by the ethical review board of the UMG (8/1/17). 

### 2.3. Cell Cultivation, Transfection, and Treatment 

Primary mouse PDAC cells were cultivated utilizing Dulbecco’s modified Eagle’s medium (DMEM) (Gibco, Thermo Fisher Scientific, Waltham, MA, USA, 41965062) containing 4.5 g/L D-Glucose, L-Glutamine, supplemented with 10% fetal calf serum (FCS) (Sigma-Aldrich, St. Louis, MI, USA, S0615) and 1% nonessential amino acids (NEAA) (Gibco, Thermo Fisher Scientific, Waltham, MA, USA, 11140035). CDX cells were cultured in Keratinocyte-SFM:RPMI (in 3:1 ratio) (Thermo Fisher Scientific, Waltham, MA, USA, 17005034; Gibco, Thermo Fisher Scientific, Waltham, MA, USA, 61870044) media supplemented with 2% FCS, 1% Penicillin-Streptomycin (Sigma-Aldrich, St. Louis, MI, USA, P0781), bovine pituitary extract (Thermo Fisher Scientific, Waltham, MA, USA 13028014), and epidermal growth factor (Sigma-Aldrich, St. Louis, MI, USA, E964). Cells were maintained at 37 °C in a humidified atmosphere with 5% (*v*/*v*) CO_2_. Mycoplasma contamination was excluded regularly. The generation of shRNA-mediated EZH2 knockdown cells has been described previously [13]. To transiently knockdown EZH2 (Ambion, Thermo Fisher Scientific, Waltham, MA, USA, AM16708, ID: 61436) or p19^Arf^ (Ambion, Thermo Fisher Scientific, Waltham, MA, USA AM16708, ID: 262856) cells were transfected with small interfering RNA (siRNA) using siLentFect lipid reagent (Bio-Rad Laboraties, Hercules, CA, USA, 170-3362) in 200 µL OptiMEM (Gibco, Thermo Fisher Scientific, Waltham, MA, USA, 31985-062). Sequences of the siRNA are listed in Supplementary Table S2. Silencer Negative Control No. 1 siRNA (Ambion, Thermo Fisher Scientific, Waltham, MA, USA, 4611) was used as the negative control. Constructs containing human p53^wt^ and p53^R175H^ were kindly gifted by Prof. Matthias Dobbelstein, UMG. For p53^wt^ and p53^R175H^ overexpression, cells were transfected with 2 µg per one 6-well of the respective construct using lipofectamine 2000 (Thermo Fisher Scientific, Waltham, MA, USA, 11668-019) in 200 µL OptiMEM. Apoptosis induction was achieved by 0.5 µM staurosporine (STS) (Cell Signaling Technology, Danvers, MA, USA, 9953) treatment for 24 h or by 10 µM 5-FU (Sigma-Aldrich, St. Louis, MI, USA, F6627) treatment for 24 h. The inhibition of the proteasome was performed by treatment with 20 µM MG132 (Merck, Darmstadt, Germany, 474790) for 1 h, and the blocking of the translation was achieved by 20 mg/mL cycloheximide (Cell Signaling Technology, Danvers, MA, USA, 2112) treatment for 7–18 min as indicated.

### 2.4. RNA Isolation and Quantitative Real-Time PCR (qRT-PCR)

Total RNA was isolated from PDAC cells using TRIzol followed by phenol-chloroform purification. Subsequently, 1 µg RNA was utilized for reverse transcription into cDNA using iScript cDNA synthesis kit (BioRad Laboraties, Hercules, CA, USA, 170-8891). qRT-PCR analyses were performed in triplicates using SYBR Green. Primer sequences are listed in Supplementary Table S3. The mRNA expression of all target genes was normalized to the housekeeping gene *Rplp0* and, additionally, to the control condition.

### 2.5. Protein Harvesting and Western Blotting

Protein isolation of PDAC cells and pancreatic tissue was performed using whole-cell lysis buffer complemented with 1× complete EDTA-free protease inhibitor cocktail (Roche Holding, Basel, Switzerland, 4693132001), phenylmethylsulfonyl fluoride (PMSF), sodium fluoride (NaF), and sodium orthovanadate (NaO), as previously described [13,29]. Bradford reagent (Bio-Rad Laboraties, Hercules, CA, USA, 5000006) was used for protein concentration determination. The procedure of Western blotting was performed as has been described previously [13]. All antibodies and their dilution used for this study are listed in Supplementary Table S4. Detected protein bands were visualized using chemiluminescence (PerkinElmer, NEL103001EA, Waltham, MA, USA) at INTAS ChemoCam imager.

### 2.6. Annexin/Propidium Iodide (PI) Staining

For measuring apoptosis induction, Annexin-V and propidium iodide staining was performed and analysed as described previously [32].

### 2.7. Immunofluorescence and Immunohistochemistry (IHC)

Murine pancreas and liver tissue were collected upon sacrificing mice and subsequently embedded in paraffin as has been described before [14]. Immunofluorescence staining in pancreatic tissue was performed as previously described [13]. P19^Arf^ antibody and its dilution are depicted in Supplementary Table S5. Images of stained tissue sections were taken with Leica LAS X software under a Leica DMi8 microscope. Positive staining was counted in six representative images of six different mice per condition using ImageJ Fiji. Hematoxylin and eosin and immunohistochemistry (IHC) staining in FFPE material were performed as previously explained [33]. All antibodies used for IHC are listed in Supplementary Table S5. For staining with a primary mouse antibody on mouse tissue (αSMA), the M.O.M. immunodetection kit (Biozol Diagnostica, Eching, Germany, BMK-2202) was used according to the manufacturer’s protocol. For Masson’s trichrome staining the stain kit from Polysciences Europe GmbH, Hirschberg an der Bergstrasse, Germany (25088) was utilized. Therefore, tissue sections were deparaffinized by washing twice in xylene for 15 min. Hydration was achieved by incubation of tissues with descending ethanol concentration (99%, 99%, 96%, 80%, 70%, 50%). Slides were incubated in Bouin’s fixative solution overnight. After washing slides for 5 min with dH_2_O sections were incubated for 15 min in mixed Weigert’s Iron Hematoxylin solution A and B followed by a washing step in dH_2_O. Subsequently, the tissue was incubated in Biebrich Scarlet-Acid Fuchsin solution for 5 min and briefly washed with water. After incubation in phosphotungstic/phosphomolybdic acid for 5 min, sections were stained with Aniline Blue for 8 min and washed in dH_2_O. Then, tissue was incubated in 1% acetic acid and washed in H_2_O prior to dehydration in ethanol (96%, 99%, 30 s each), incubation in xylene for 1 min, and mounting. The quantification of (immuno-)histochemistry was performed in ten representative images of each section by measuring positive stained areas or cells, respectively, using ImageJ Fiji. Patients were classified in EZH2^high/low^ and p14^Arf high/low^ according to their expression based on IHC staining in PDAC tissue. EZH2^low^ was defined as <7% positive EZH2 staining and p14^ARF low^ as <13% positive p14^ARF^ staining.

### 2.8. Chromatin Immunoprecipitation (ChIP) and RNA-Sequencing 

ChIP analysis was performed as described in detail elsewhere [13,32]. Briefly, cells were fixed utilizing 1% formaldehyde in PBS for 20 min prior to quenching the reaction by adding 1.25 mol/L glycine for 5 min. All antibodies used for ChIP (2 µg) are depicted in Supplementary Table S6. All primers utilized for qRT-RCR following ChIP are listed in Supplementary Table S7. ChIP analysis was performed with three biological and three technical replicates each. RNA-sequencing (RNA-seq) analysis was performed as previously described [13,32]. SiRNA-mediated EZH2 knockdown was performed in triplicates in KC, KPC, and KNPC cells. Library preparation of 500 ng of total RNA was performed using the True seq (Illumina, San Diego, CA, USA, RS-122-2001, RS-122-2002) RNA library preparation kit according to the manufacturer´s manual following cDNA library concentration determination (Qubit; Thermo Fisher Scientific, Waltham, MA, USA, Q32854) and fragment size control (Bioanalyzer; Agilent Technologies, Santa Clara, CA, USA 2100, 5067–4626). Sequencing was performed by the NGS Integrative Genomics Core Unit (NIG) of the UMG. The accession number for RNA-seq data files is GSE197006. Additionally, publicly available shRNA-mediated EZH2 knockdown RNA-seq data was utilized (GSE153491). The open-source platform Galaxy [34] (https://usegalaxy.org/, accessed on 24 May 2020) was used to analyse FastQ files. The murine transcriptome mm9 was utilized to align the reads using TopHat2 (version 2.1.0) [35]. Fragment Per Kilobase Million (FPKM) values were determined using Cuffnorm (version 2.2.1.1) [36] and Cuffdiff (version 2.2.1) [37]. To decrease background signals, genes with FPKM values < 0.01 were eliminated from the analysis, implying approximately 65% of the mouse genome. Genes were considered as significantly differentially regulated with a log2fold change of <−0.5 and >0.5 and a *q* value of < 0.05. To assess similarities of replicates principal component analysis (PCA) and sample-to-sample distances, analysis were performed in R (version 4.0.0, R Studio Team, Boston, MA, USA) using read counts resulting from HTSeq (version 0.9.1) [38]. Gene set enrichment analysis (GSEA) of our data in the indicated publicly available gene sets was performed using Signal2Noise metric for gene ranking. A threshold of 0.05 for FDR *q* values is defined as significant. Gene Ontology (GO) analysis was achieved using the EnrichR analysis tool (https://maayanlab.cloud/Enrichr/, accessed on 16 July 2021). Heatmaps were created with log10 values of FPKM data utilizing pheatmap function in R (version 4.0.0, R Studio Team, Boston, MA, USA). Venn diagrams were generated with Bioinformatics Evolutionary Genomics (http://bioinformatics.psb.ugent.be/webtools/Venn/, accessed on 16 July 2021).

### 2.9. Statistical Analysis

Data are represented as mean ± standard deviation (SD) and were visualized using GraphPad PRISM version 8.0.2 (Graphpad Software). Significance was tested using the respective statistical tests stated in the figure legends. Significance is indicated as * *p* ≤ 0.05; ** *p* ≤ 0.01; *** *p* ≤ 0.001; **** *p* ≤ 0.0001; and ns (non-significant).

## 3. Results

### 3.1. EZH2 Depletion Does Not Reduce Tumor Progression in Orthotopic PDAC Models 

**Figure 1:**
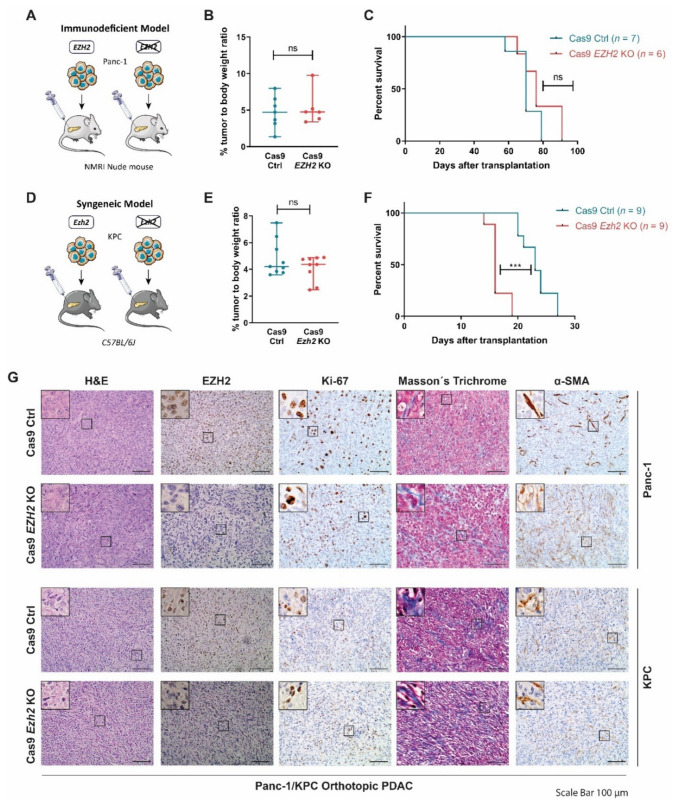
**EZH2 depletion is not beneficial in orthotopic PDAC models.** (**A**) Schematic illustration of orthotopically transplanted *EZH2* expressing (Cas9 Ctrl) and CRISPR/Cas9-mediated *EZH2* knockout (Cas9 *EZH2* KO) Panc-1 cells into immunodeficient *NMRI-Foxn1nu* (NMRI nude) mice. *EZH2* knockout was verified prior to transplantation [13,14]. (**B**,**C**) Relative tumor weight (**B**) and the Kaplan–Meier survival curve (**C**) of NMRI nude mice after orthotopic transplantation of Panc-1 cells (median survival: Cas9 Ctrl cohort: 70 days, Cas9 *EZH2* KO cohort: 76 days post transplantation). (**D**) Schematic illustration of orthotopically transplanted *Ezh2* expressing (Cas9 Ctrl) and CRISPR/Cas9-mediated *Ezh2* knockout (Cas9 *Ezh2* KO) Kras^G12D^;Trp53^R172H/+^ (KPC) cells into immunocompetent *C57BL/6J* mice for the generation of a syngeneic model. *Ezh2* knockout was verified prior to transplantation [13]. (**E**,**F**) Relative tumor weight (**E**) and Kaplan–Meier survival curve (**F**) of *C57BL/6J* mice after orthotopic transplantation of KPC cells (median survival: Cas9 ctrl cohort: 23 days, Cas9 *Ezh2* KO cohort: 16 days post transplantation). (**G**) Representative images of hematoxylin and eosin (H&E), Masson’s trichrome, and indicated immunohistochemistry stainings in orthotopic PDAC. Scale bar, 100 µm. (**B**,**E**) Each dot represents one mouse, values represent mean ± SD, two-tailed unpaired Student’s *t*-test, ns, non-significant. (**C**,**F**) Significance was determined by Log-rank (Mantel–Cox) test, ***, *p* ≤ 0.001, ns, non-significant.).

In order to address the relevance of EZH2 in PDAC progression and aggressiveness, we orthotopically transplanted either wildtype EZH2 (Cas9 Ctrl)- or *EZH2*-depeleted (Cas9 *EZH2* KO) Panc-1 cells into *NMRI-Foxn1nu* (further referred to as NMRI Nude) mice (Figure 1A) [13,14]. Of note, interference with EZH2 expression reduced clonogenicity and proliferation of *EZH2*-deficient Panc-1 cells in vitro [13,14]. Surprisingly, however, we neither detected a reduced relative tumor weight nor prolonged survival in the absence of *EZH2* (Figure 1B,C). Given the reported functional relevance of the immune environment for PDAC progression [23,39,40,41], we complemented our immunodeficient in vivo study with a syngeneic immunocompetent orthotopic transplantation model. To this end, we generated *Ezh2* knockout cells by applying CRISPR/Cas9-based genome-editing to primary PDAC cells derived from the well-established *Kras^G12D^;Trp53^R172H/+^* (*KPC*) model [13], which combines pancreas-specific (pdx1-Cre) oncogenic Kras activation and a gain-of-function *Trp53* mutation [13,27] and subsequently orthotopically transplanted Cas9 Ctrl and Cas9 *Ezh2* KO cells into immune-competent *C57BL/6J* mice (Figure 1D). Comparable with our findings in the Panc-1 transplantation model (Figure 1B,C), *Ezh2*-deficiency did not affect the pancreatic tumor weight of the recipient mice (Figure 1E) and the survival of the Cas9 *Ezh2* KO transplanted mice was even reduced compared to Cas9 Ctrl animals (Figure 1F). Consistent with the observed phenotypes, the loss of EZH2 expression in both orthotopic models neither reduced tumor cell proliferation (as determined by the immunohistochemical analysis of Ki67, Figure 1G and Figure S1A), nor did it render the composition of the PDAC stroma less aggressive, as illustrated by the determination of the activated stroma index (αSMA-positive fibroblasts/collagen) [42] (Figure 1G and Figure S1B,D).

Together, in contrast to previous results suggesting a strong tumor-promoting implication of the methyltransferase in PDAC [12,13,14], our findings in human and murine orthotopic PDAC models suggest that *EZH2* depletion is not necessarily sufficient to block tumor progression in favour of a less aggressive PDAC phenotype. 

### 3.2. The TP53-Status Determines EZH2-Dependent Target Gene Regulation

**Figure 2:**
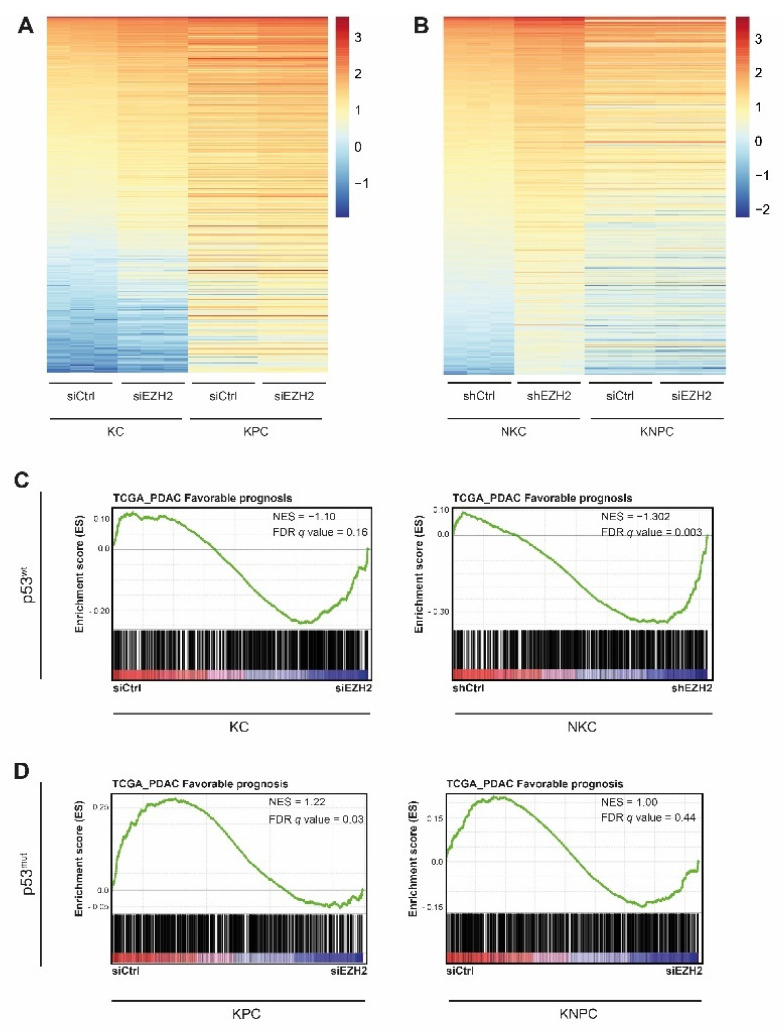

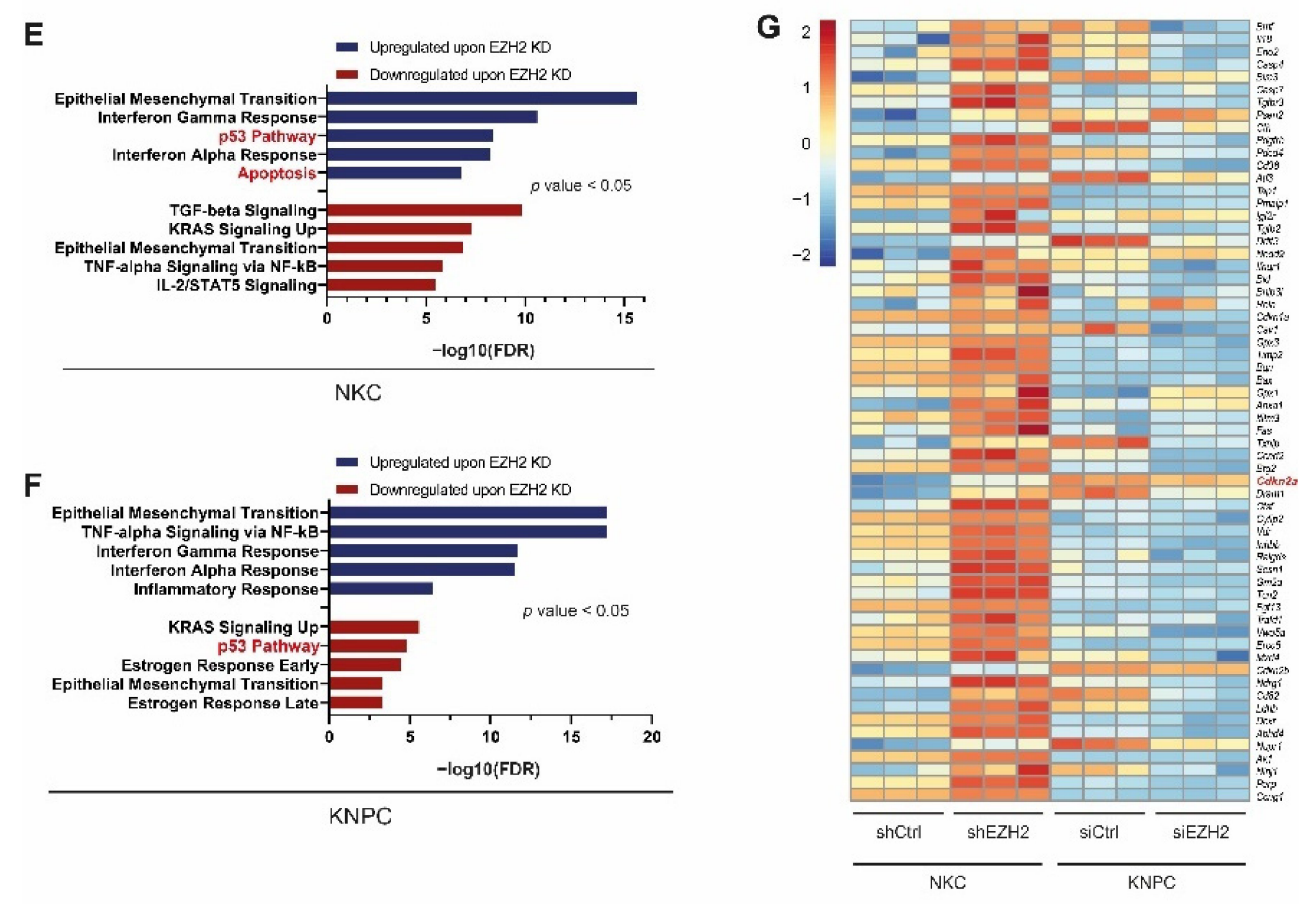
***TP53*-status determines EZH2-dependent target gene regulation.** (**A**,**B**) Heatmap illustrating expression of genes with significant upregulation (FPKM > 0.01, log2FC > 0.5, *q* < 0.05) upon knockdown of EZH2 in the indicated p53wt cells (*Kras^G12D^* (KC): 384 upregulated genes) and *caNFATc1;Kras^G12D^* (NKC): 1322 upregulated genes) and its consequences on the expression of these genes in the respective p53mut cells (*Kras^G12D^;Trp53^R172H/+^* (KPC) and *Kras^G12D^;caNFATc1;Trp53^R172H/+^* (KNPC)). (**C**,**D**) Gene set enrichment analysis (GSEA) in the indicated p53wt (**C**) and p53mut (**D**) PDAC cells comparing the enrichment of ‘favorable prognosis’-associated genes (Supplementary Table S8) [45] upon knockdown of EZH2 after RNA-sequencing. Normalized enrichment score (NES) and (false discovery rate) FDR as indicated in the graph (GSEA of NKC as previously shown in [13]). (**E**,**F**) Gene ontology (GO) analysis to reveal significantly up- or downregulated pathways upon EZH2 depletion in the indicated PDAC cells (*p* < 0.05). (**G**) Heatmap demonstrating expression of 62 selected pro-apoptotic and/or p53-pathway associated genes in NKC and KNPC cells upon EZH2 knockdown after RNA-seq (FPKM > 0.01).

The activity, target gene regulation, and biological function of epigenetic regulators in general and of EZH2, in particular, underlay a strong context-dependency [24]. This is, for instance, reflected in the tissue-specific involvement of the histone methyltransferase in tumor-promoting (e.g., PDAC, glioblastoma [15,43]) vs. tumor-suppressive (e.g., colorectal cancer [44]) programs. However, even within the same tumor entity, the molecular makeup of a tumor cell and/or hierarchical signalling cues converging on EZH2 significantly determine EZH2-dependent target gene regulation and hence critically affect the functional implications of the methyltransferase [12,13,14]. Consequently, we asked whether molecular characteristics evident in both the Panc-1 and the KPC orthotopic model account for the unforeseen outcome of our in vivo studies. The major difference between the previously utilized PDAC model systems, in which we observed a tumor-promoting role of EZH2 [12,13], and the herein described mice is the *TP53*-status. To explore, whether the *TP53*-status indeed directs the contrary functional implications of EZH2 depletion observed in PDAC, we took advantage of four different primary PDAC cell lines generated either from *Trp53* wildtype (further referred to as p53wt) or *Trp53 ^R172H/+^* (resulting in a gain-of-function p53 mutation, p53mut) transgenic PDAC models. P53wt PDAC cells were obtained from the well-established *Kras^G12D^* (KC) [26] and the *caNFATc1;Kras^G12D^* (NKC) [28] genetically engineered mouse models, while cells representing p53mut PDAC were derived from the aforementioned KPC [27] and the *Kras^G12D^;caNFATc1;Trp53^R172H/+^* (KNPC) [30] transgenic models. We subjected those four PDAC cell lines to *Ezh2* knock-down using siRNA (KC, KPC, KNPC) and shRNA (NKC) [13] technology (Supplementary Figure S2A) and performed RNA-seq analysis to examine global *Trp53*-status-dependent transcriptional consequences of EZH2 blockade. For NKC cells, we used RNA-seq raw data, which has been published previously [13] but was analysed using the same pipeline as for the KC, KPC, and KNPC cells. Principle Component Analysis (PCA) and sample-to-sample distances confirmed the similarity of triplicates and revealed four different conditions (Supplementary Figure S2B–F). To assess whether EZH2-dependent gene regulation differs in p53wt and p53mut PDAC, we first filtered for genes which were significantly upregulated upon EZH2 knockdown in KC- and NKC cells (FPKM > 0.01; log2fold change > 0.5; *q* value < 0.05; KC: 384 genes, NKC: 1322 genes). Interestingly, the expression of genes which were found to be upregulated upon EZH2 knockdown in p53wt cells remained predominantly unchanged in the respective p53mut counterparts (Figure 2A,B). To consider also PRC2-independent EZH2 activities, we also explored the impact of the *Trp53* status on genes downregulated upon EZH2 knockdown (FPKM > 0.01; log2fold change < −0.5; *q* value < 0.05, KC: 188 genes, NKC: 964 genes). Consistent with the set of upregulated genes, the expression of EZH2 targets downregulated in p53wt cells remained largely stable in KPC and KNPC cells (Supplementary Figure S2G,H), thus pointing towards distinct EZH2-dependent gene regulation programs in the presence and absence of p53mut.

Next, we aimed to explore, whether the *Trp53*-status impacts the EZH2-dependent transcription programs with potential functional implications in tumor progression and disease outcome. Consistent with our previous findings [13], the integration of our RNA-seq data with publicly available transcriptome data [45] revealed a significant enrichment of ‘favorable prognosis genes’ (Supplementary Table S8) in EZH2-depleted vs. siCtrl NKC cells and a reasonable enrichment in KC cells (Figure 2C). However, in the context of p53mut, the gene set associated with a favorable PDAC prognosis was either unaffected by EZH2 (KNPC) or even associated with the presence of EZH2 (KPC) (Figure 2D), thus reflecting the survival data in our p53mut in vivo models (Figure 1). To examine additional differences in *Trp53*-status determined EZH2-dependent target gene regulation we performed GO analysis. Interestingly, in p53wt PDAC cells EZH2 knockdown resulted in the enrichment of both apoptosis- and p53-related pathways (Figure 2E and Figure S2I). In contrast, in p53mut cells, these pathways were not enriched upon EZH2 knockdown or even downregulated as observed in KNPC cells (Figure 2F and Figure S2J). Consistently, the expression of 62 genes selected based on their implication in pro-apoptotic processes and/or p53-signaling pathways was only found to be upregulated upon EZH2 knockdown in NKC-, but not in KNPC cells (Figure 2G). 

Together, our findings suggest that the *Trp53*-status critically affects EZH2-dependent target gene regulation and indicates that the induction of p53- and apoptosis-related gene signatures upon EZH2 knockdown is restricted to p53wt PDAC. 

### 3.3. The Induction of PDAC Cell Apoptosis upon EZH2 Depletion Is Restricted to p53wt Status

**Figure 3:**
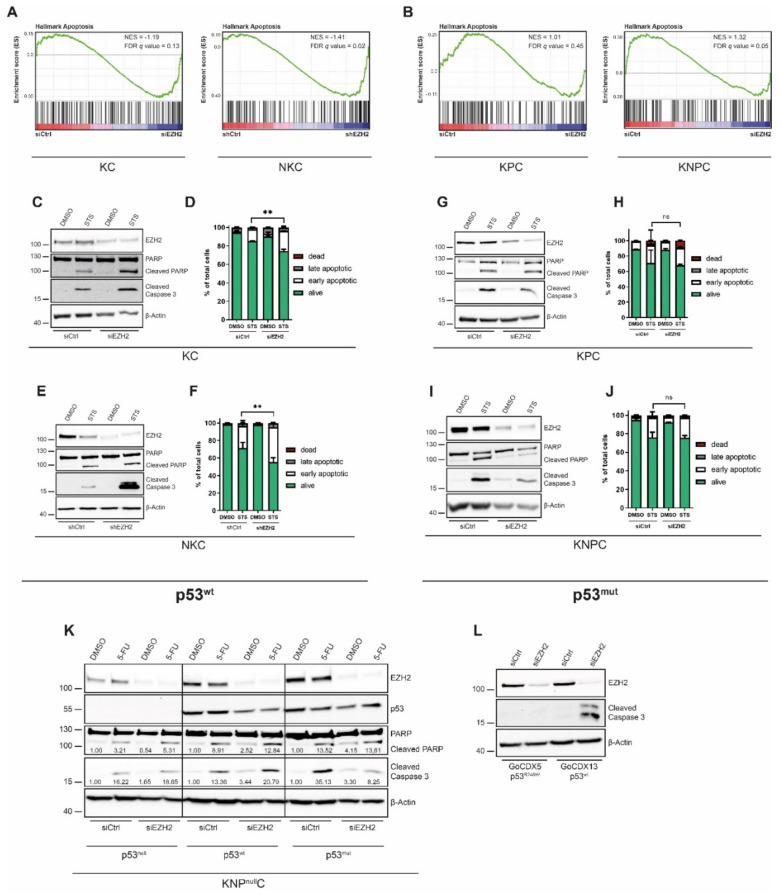
**Inverse effects of apoptotic processes depending on the *Trp53*-status.** (**A**,**B**) GSEA plot in the indicated p53wt (**A**) and p53mut (**B**) PDAC cells comparing the enrichment of apoptosis-related genes upon knockdown of EZH2 after RNA-seq. NES and FDR as indicated on the graph. (**C**–**J**) Depletion of EZH2 in PDAC cells with p53wt (KC, NKC) and p53mut (KPC, KNPC) expression and simultaneous treatment with staurosporine (STS). Western blot analysis of apoptosis-related proteins (**C**,**E**,**G**,**I**) and Annexin-V/propidium iodide staining and subsequent FACS analysis ((**D**,**F**,**H**,**J**), *n* = 3)) in KC, NKC, KPC, KNPC cells. Significance was determined by Student’s *t*-test; **, *p* ≤ 0.01; ns, non-significant. (**K**) Western blot analysis of apoptosis-related proteins upon knockdown of EZH2 and transfection with p53^wt^ and p53^mut^ constructs, respectively, together with treatment with 5-FU in Kras^G12D^;caNFATc1;Trp53^fl/fl^ (KNP^null^C) cells. The densitometric quantification of cleaved PARP and cleaved caspase 3 detection was performed using ImageJ and is revealed under the respective band. The band intensities are normalized to the respective siCtrl DMSO condition with the same p53-status, which have been set to 1.00. (**L**) Western blot analysis in human primary PDAC cells with p53wt (GöCDX13) and p53mut (GöCDX5) expression upon knockdown of EZH2.

Next, we aimed at exploring the efficacy and the *Trp53* status-specificity of EZH2 knockdown for the induction of pro-apoptotic cellular programs. Consistent with our GO analysis (Figure 2E,F and Figure S2I,J) the ‘Hallmark Apoptosis’ gene set was found to be significantly enriched in EZH2-depleted p53wt cells (Figure 3A), while GSEA in p53mut cells revealed the same signature to be unaltered (KPC) or even to be enriched in the siCtrl condition (KNPC) (Figure 3B). In order to examine the *Trp53*-status-dependent consequences of EZH2 knockdown on PDAC cell apoptosis at the functional level, we investigated PARP cleavage and cleaved Caspase 3 expression in the presence and absence of EZH2 and conducted Annexin-V staining and subsequent FACS analysis to assess the percentage of apoptotic cells. Given that PDAC cells have a very low basal apoptotic propensity, we utilized the potent protein kinase C inhibitor staurosporine (STS) as a tool to induce apoptosis [46]. Importantly, STS treatment combined with EZH2 knockdown effectively enhanced PARP cleavage and cleaved Caspase 3 expression in NKC and KC cells and increased the fraction of early and late apoptotic cells (Figure 3C–F and Figure S3A). In contrast, PARP cleavage and cleaved Caspase 3 expression remained stable or even decreased upon EZH2 knockdown in STS-treated KPC and KNPC cells, and no changes in the apoptotic cell fraction were detected via Annexin-V staining in p53mut cells (Figure 3G–J and Figure S3A). To confirm the relevance of the *Trp53wt* status for cleavage of Caspase 3 and PARP upon EZH2 knockdown, we utilized an additional tool for apoptosis induction and treated PDAC cells with the chemotherapeutic agent 5-FU. In line with our previous findings, EZH2 knockdown combined with 5-FU treatment considerably increased cleaved Caspase 3 expression and PARP cleavage in p53wt cells (Supplementary Figure S3B,D), while EZH2 depletion did not affect the 5-FU-dependent expression of the same pro-apoptotic proteins in p53mut cells (Supplementary Figure S3E,F). To exclude, that the distinct EZH2-dependent regulation of apoptosis was caused by cell line-intrinsic characteristics other than the *Trp53*-status, we took advantage of a p53null system (*Kras^G12D^;caNFATc1;Trp53^Δ/Δ^*, KNP^null^C cells [30]), re-expressed p53wt or p53mut constructs alongside with EZH2 knockdown and simultaneously treated with 5-FU. In the p53null condition, EZH2 knockdown did not alter PARP cleavage or cleaved Caspase 3 expression (Figure 3K). However, upon re-expression of p53wt, but not in the context of p53mut, EZH2 knockdown strongly increased 5-FU-induced PARP and Caspase 3 cleavage (Figure 3K), emphasizing that the EZH2-dependent regulation of pro-apoptotic processes is indeed p53-status-dependent. Consistently, EZH2 knockdown increased cleaved Caspase 3 expression only in p53wt- (GöCDX13), but not in p53mut (GöCDX5, *TP53^R248W^*) human primary PDAC cells (Figure 3L), suggesting that the impact of the *TP53*-status on EZH2-dependent regulation of pro-apoptotic processes is conserved in human PDAC.

Hence, our in vitro findings highlight the *TP53*-status as a crucial context-determining cue in directing the functional consequences of EZH2 blockade and suggest that sufficient induction of PDAC cell apoptosis upon EZH2 depletion is restricted to p53wt PDAC.

### 3.4. Loss of EZH2 Results in p53wt Stabilization in a CDKN2A-Dependent Manner

**Figure 4:**
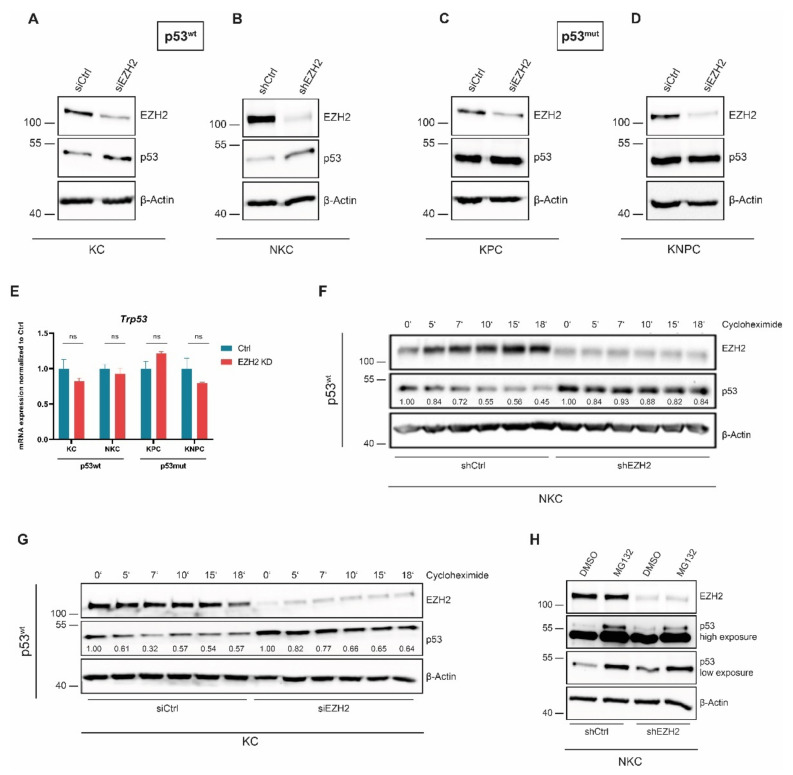
**EZH2 controls p53wt on a post-translational level.** (**A**–**E**) Western blot analysis (**A**–**D**) and qRT-PCR analysis (*n* = 3) € in the indicated p53wt and p53mut cells upon knockdown of EZH2. Values represent mean ± SD. Significance was determined by Student’s *t*-test; ns, non-significant. (**F**,**G**) Western blot analysis in the indicated p53wt cells upon short-term treatment (0–18 min) with cycloheximide to block translation and visualize the difference in p53 half-life between EZH2-proficient and -deficient cells. Densitometric quantification of p53 detection was performed using ImageJ and is revealed under the respective band. The p53 band intensities determined at the different time-points of cycloheximide treatment are normalized to the respective basal (0′) p53 levels, which have been set to 1.00. (**H**) Short-term treatment with the proteasome inhibitor MG132 and subsequent western blot analysis to analyze ubiquitination of p53.

Given that our transcriptome analysis revealed differential and *Trp53*-status-dependent gene regulation upon EZH2 depletion for p53-dependent gene sets as well (Figure 2E–G and Figure S2I,J), we sought to explore the impact of EZH2 activity on p53 expression and protein-turnover. Interestingly, EZH2 knockdown strongly increased p53 protein expression in p53wt PDAC cells (Figure 4A,B and Figure S4A). In contrast, p53mut remained unaltered upon interfering with EZH2 expression (Figure 4C,D). Given that we could not detect any alterations of *Trp53* mRNA expression upon EZH2 knockdown regardless of the *Trp53* mutation status (Figure 4E), we hypothesized that EZH2 interferes with p53 expression at the posttranslational level. In unstressed cells, the rapid proteasomal degradation of p53wt is mediated by posttranslational p53 ubiquitination installed by E3-ligases, such as Mouse Double Minute 2 homolog (MDM2) [47,48]. In contrast to the physiological regulation of p53wt, *TP53* mutations, in particular gain-of-function mutations, render the p53 protein unsusceptible to MDM2-dependent destabilization [49,50]. To test whether EZH2 knockdown impacts p53wt protein stability, we blocked de novo p53 protein translation with cycloheximide and subsequently investigated the half-life of the p53 protein. Interestingly, EZH2 depletion slowed down p53 degradation both in NKC and in KC cell (Figure 4F,G). Accordingly, short-term treatment with the proteasome inhibitor MG132, which blocks proteasomal p53 degradation and hence also facilitates the detection of ubiquitinated p53 [51], revealed a higher p53 ubiquitination in the presence of EZH2 (Figure 4H), suggesting that EZH2 downregulates p53wt expression by promoting p53 degradation. 

**Figure 5:**
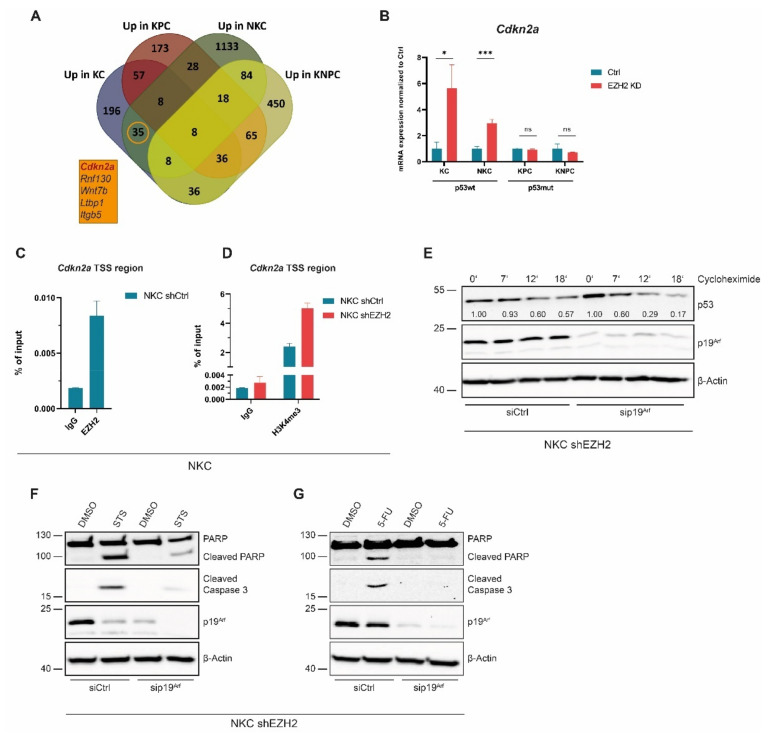
**EZH2 regulates p53 via *Cdkn2a*.** (**A**) Venn diagram showing the overlay of significantly upregulated (FPKM > 0.01, log2FC > 0.5, *q* < 0.05) genes in the indicated PDAC cells upon knockdown of EZH2. Box depicts an excerpt of five exemplary genes of the 35 genes that are only upregulated in p53wt but not in p53mut PDAC cells. (**B**) *Cdkn2a* expression in the indicated PDAC cells upon EZH2 depletion analysed by qRT-PCR (*n* = 3). Values represent mean ± SD. Significance was determined by Student’s *t*-test; *, *p* ≤ 0.05; ***, *p* ≤ 0.001; ns, non-significant. (**C**,**D**) ChIP and subsequent qRT-PCR in NKC cells displaying occupancy of EZH2 at the *Cdkn2a* TSS region (**C**) and increased H3K4me3 occupancy at the *Cdkn2a* gene (**D**) (*n* = 3). (**E**) Western blot analysis in NKC shEZH2 cells upon knockdown of p19^Arf^ and short-term treatment (0–18 min) with cycloheximide to reveal the influence of p19^Arf^ on p53 half-life. The densitometric quantification of p53 detection was performed using ImageJ and is depicted under the respective band. The p53 band intensities determined at the different time-points of cycloheximide treatment are normalized to the respective basal (0′) p53 levels, which have been set to 1.00. (**F**,**G**) Knockdown of p19^Arf^ in NKC shEZH2 cells and simultaneous treatment with STS (**F**) and 5-FU (**G**) followed by Western blot analysis.

MDM2-dependent p53 degradation underlies tight regulation by p14^ARF^, which inhibits the E3-ligase activity by complex formation, thus stabilizing p53wt expression [52]. Interestingly, we detected the *Cdkn2a* gene, which encodes for the mouse homolog of p14^ARF^, p19^Arf^*,* among the apoptosis- and p53-signature involved genes which were up-regulated upon EZH2 knockdown in NKC, but not in KNPC cells (Figure 2G). Moreover, *Cdkn2a* displayed also one of 35 target genes, which were restrictively upregulated upon EZH2 blockade in p53wt-, but not in p53mut cells (Figure 5A and Figure S4B and Supplementary Table S9). *Trp53*-status distinct EZH2-dependent mRNA-expression of a selection of these 35 target genes, including *Cdkn2a*, was validated in independent experiments (Figure 5B and Figure S4C–G). Consistently, the higher expression of *Cdkn2a* upon EZH2 knockdown also resulted in higher p19^Arf^ protein level (Supplementary Figure S4A). In accordance with previous results [53,54,55], ChIP experiments conducted in NKC cells revealed a strong binding of EZH2 at the transcriptional start site (TSS) of the *Cdkn2a* gene (Figure 5C), suggesting that *Cdkn2a* represents a direct EZH2 target. Consistent with the increase of *Cdkn2a* transcription upon EZH2 knockdown, we detected increased occupancy of the promoter/TSS activity-indicating H3K4me3 histone mark [56] upon EZH2 knockdown (Figure 5D). To explore, whether the regulation of *Cdkn2a* expression is indeed causatively involved in EZH2-dependent p53 destabilization, we studied the impact of p19^Arf^ knockdown on the half-life of p53 in EZH2-deficient NKC cells. As shown in Figure 5E, p53 degradation was remarkably accelerated in the absence of p19^Arf^, indicating that *Cdkn2a* expression is crucial for EZH2-dependent regulation of p53wt stability. Consistently, knockdown of p19^Arf^ hampered STS- or 5-FU-induced apoptosis induction in the absence of EZH2 (Figure 5F,G).

Together, these data illustrate that EZH2-dependent control of *Cdkn2a* transcription is essential for the EZH2-mediated p53wt destabilization and blockade of apoptosis programs. 

### 3.5. P53wt PDAC Evolving in the Absence of EZH2 Circumvents Induction of the CDKN2A-p53wt Axis

**Figure 6:**
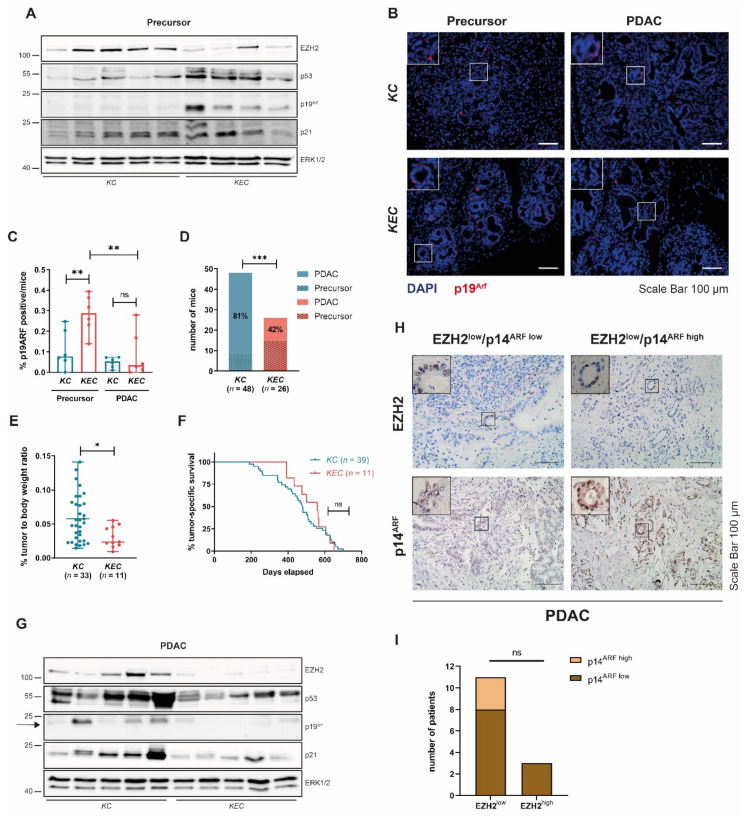
**EZH2-independent p19^Arf^ regulation in vivo**. (**A**) Western blot analysis with pancreatic lysates derived from 8-week-old *KC* and *KEC* mice carrying PDAC precursor lesions. (**B**) Representative images of immunofluorescence staining of p19^Arf^ in precursor (12 week) and PDAC of *KC* and *KEC* mice. Scale bar, 100 µm. (**C**) Quantification of p19^Arf^ staining. Counting was performed in six representative images of six different mice per condition using ImageJ Fiji. One dot represents one mouse. Significance was determined using two-tailed unpaired Student’s *t*-test, *, *p* ≤ 0.05; **, *p* ≤ 0.01, ns, non-significant. (**D**,**E**) Bar graph showing tumor incidence (significance was determined using Fisher’s exact test, ***, *p* ≤ 0.001) (**D**) and relative body weight (each dot represents one mouse, significance was determined using two-tailed unpaired Student’s *t*-test, *, *p* ≤ 0.05) (**E**). (**F**) Kaplan–Meier plot of *KC* and *KEC* mice to illustrate tumor-specific survival. Significance was determined by log-rank (Mantel–Cox) test. ns, non-significant. (**G**) Western blot analysis from PDAC lysates derived from *KC* and *KEC* mice. Arrow indicates an unspecific band. (**H**) Representative images of EZH2 and p14^ARF^ immunohistochemistry of human PDAC grouped in EZH2^low^/p14^ARF low^ and EZH2^low^/p14^ARF high^. Scale bar, 100 µm. (**I**) Bar graph showing quantification of p14^ARF^ positive cells in human PDAC (**H**). Significance was determined using two-tailed unpaired Student’s *t*-test, ns, non-significant.

To validate the functional relevance of EZH2-dependent repression of the p19^Arf^-p53wt-axis for EZH2-dependent PDAC development and progression in vivo, we took advantage of a p53wt transgenic PDAC model, which combines pancreas-specific *Ezh2*-deficiency with oncogenic Kras activation (*Kras^G12D^;Ezh^fl/fl^* (*KEC*) mice [8,14]. Compared to EZH2-proficient *KC* mice, *KEC* mice display a reduced number and severeness of PDAC precursor lesions [14]. Consistent with our hypothesis that EZH2 represses the p19^Arf^-p53wt axis, we observed increased p19^Arf^ and p53 expression in *KEC* vs. *KC* PDAC precursor lesions (Figure 6A–C), suggesting that in the context of *Ezh2* deficiency oncogene-induced failsafe programs are at least partially maintained in the *Kras* mutant pancreas. Accordingly, we found a significantly reduced tumor incidence of *KEC-* compared to *KC* mice (42 vs. 81%, Figure 6D) and pancreatic tumors that did form in the absence of *Ezh2* showed a reduced relative tumor weight (Figure 6E). However, the tumor-specific survival of *KC* and *KEC* mice did not differ (Figure 6F), suggesting that PDAC that does form in the absence of EZH2 displays a similar aggressiveness as EZH2-proficient tumors. Consistent with this observation and in contrast to our analysis in pancreatic precursor lesions, we detected only very low p19^Arf^ expression in PDAC tissue and did not observe an increase of p19^Arf^ in *KEC* vs. *KC* tumors (Figure 6B,C,G). Consistently, the expression of p53 and its downstream target p21 was not increased in *KEC* vs. *KC* PDAC (Figure 6G), suggesting that PDAC that evolves despite the absence of *Ezh2* bypasses upregulation of p19^Arf^ expression and subsequent stabilization of p53wt in favour of a progressive tumor phenotype. To explore, whether hampered p14^ARF^ expression also occurs in human p53wt PDAC subtypes characterized by low EZH2 expression, we took advantage of a collection of tumor specimens obtained from resected PDAC patients. From this cohort, we selected all tumors which are characterized by wildtype expression of both *TP53* and *CDKN2A* (*n* = 14, identified by gene-panel-sequencing) and subsequently performed EZH2 and p14^ARF^ immunohistochemistry to group specimens into EZH2^high/low^ and p14^ARF high/low^ cases (Figure 6H and Figure S4H). In line with previous findings linking high EZH2 levels and activity to advanced PDAC disease and dedifferentiation [13,15], EZH2 expression in our cohort of resected PDAC patients was comparably low. However, consistent with the role of EZH2 in transcriptional *CDKN2A* repression, all EZH2^high^-grouped PDAC samples displayed low p14^ARF^ expression (Figure 6I and Figure S4H). Interestingly, although EZH2 expression was nearly undetectable in the EZH2^low^ group, p14^ARF^ expression remained as low as in the EZH2^high^ group in 8/11 EZH2^low^ PDAC samples (Figure 6I and Figure S4F). In the absence of genetic *CDKN2A* alterations in this cohort, these findings point towards EZH2-independent epigenetic mechanisms blocking *CDKN2A*/p14^ARF^ expression in these tumors.

In summary, our findings reveal a hitherto unknown p53-status dependency of EZH2 function in PDAC and implicate that the therapeutic efficacy of pharmacological interference with EZH2 might be restricted to PDAC subtypes with a functional *CDKN2A*-p53wt axis. 

## 4. Discussion

As a consequence of the multiple cell-intrinsic and -extrinsic cues converging in the expression and activity of epigenetic regulatory proteins in cancer, the same epigenetic mechanisms occurring in different contexts can result in multifaceted and partially diverse functional outcomes. This is, for instance, reflected in observations linking high activity of specific epigenetic regulatory proteins with tumor-suppressive functions in one group of malignancies, while the same protein promotes tumor progression in other tumor entities [24,57,58,59]. Those tissue-specific oncogenic implications have been also reported for EZH2. While the methyltransferase has been associated with oncogenic properties in, e.g., lung- and prostate cancer [60,61], glioblastoma [43], or lymphoma [1], tumor-suppressive EZH2 activities have been revealed for T-cell acute lymphoblastic leukemia [62], clear cell renal carcinoma [63], or colorectal cancer [44]. However, antithetical EZH2 functions do not only occur among different tumor entities but are also evident within one tissue type. Constitutive active KRAS signaling, for instance, switches tumor-suppressive EZH2 functions evident in early pancreatic carcinogenesis into tumor-progressive activities in advanced neoplasia. Mechanistically, the *KRAS* status determines the transcriptional consequences of EZH2-dependent targeting of the *NFATc1* gene, encoding for an inflammatory tumor-progressive transcription factor [28,64]. While EZH2 represses *NFATc1* in the *KRAS* wildtype context, thus counteracting pancreatic metaplasia, EZH2 activity leads to the transcriptional activation of the same gene in the *KRAS* mutant pancreatic cell, thus promoting oncogenic NFATc1-activity and PDAC development [14]. Herein, we demonstrate opposing EZH2 activities even in established PDAC and identify the mutation of the master tumor suppressor gene *TP53* as the context-defining molecular event determining EZH2-controlled gene regulation and tumor progression in PDAC maintenance. *TP53* mutations are well-characterized for their impact on the regulation of tumor failsafe mechanisms [65,66,67], metabolism [68], and immune cell infiltration [69] and are detected in more than 70% of PDAC specimens, particularly in poorly differentiated basal-like PDAC subtypes [17]. Importantly, p53 proteins characterized by missense mutations modulate gene transcription by modifying the activity or target gene selection of co-transcription factors in many cancer entities, including PDAC [30,70,71]. Consistent with the pivotal implications of p53 on transcriptional reprogramming processes, we illustrate that the p53 status determines the consequences of EZH2 depletion on the induction of apoptosis-related gene signatures and cell programs and defines the prognostic impact of interfering with EZH2 expression in PDAC. A p53-status-determined prognostic relevance of interfering with PRC2 activity has been previously reported in Non-Small-Cell-Lung-Cancer (NSCLC) models, where PRC2 inhibition was efficient to prevent NSCLC formation in p53wt, but not in the p53mut context [72]. In line with these reports, our in vivo findings suggest that the therapeutic efficacy of interfering with EZH2 expression or activity might be restricted to PDAC subtypes with an intact *CDKN2A*-p53wt PDAC axis, thus emphasizing the significance of molecular stratification for successful implementation of EZH2-blocking therapeutic strategies in PDAC treatment.

The multifarious functional involvement of EZH2 in physiological and pathological conditions including cancer is potentiated by the existence of both canonical- and non-canonical EZH2 activities. In the canonical EZH2 pathway, the histone methyltransferase acts within the PRC2 complex and trimethylates H3K27, thus controlling transcriptional repression. However, EZH2 also modulates gene expression in a PRC2-independent manner [73]. Interestingly, PRC2-independent EZH2 activities have been linked with transcriptional activation rather than H3K27me3-mediated repression and regularly involve the recruitment or binding of non-PRC2-proteins. In glioblastoma, for instance, EZH2 interacts with and post-translationally methylates the tumor-promoting inflammatory transcription factor STAT3, thus increasing its transcriptional activity [43]. In breast and prostate cancer, EZH2 drives transcriptional activation by interacting with NF-κB [74] and the androgen receptor [60], respectively, and recent findings suggest that EZH2-cMYC complexes co-activate joint gene signatures, thus promoting acute leukemia [73]. Its ability to form non-PRC2 complexes significantly broadens the spectrum of target genes potentially controlled by EZH2 and might at least partially explain the multiple and controversial EZH2 functions described in cancer. Herein, we reveal a canonical mechanism contributing to the EZH2-dependent destabilization of p53wt, which requires the transcriptional repression of *CDKN2A*. However, in light of previous reports revealing a physical interaction of EZH2 and the p53 antagonist MDM2 [75] and recent findings suggesting EZH2 as a specific and important inducer of the translation of the p53 mutant protein in prostate cancer [76], non-canonical EZH2 functions might additionally contribute to the increased protein turnover of p53wt in EZH2-proficient PDAC. Evidence for non-canonical EZH2 activity also exists for PDAC, where EZH2 forms a biochemical complex with the NFATc1 transcription factor. Interestingly, the complex is not involved in joint target gene regulation, hinting towards the chromatin-independent activities of the EZH2-NFATc1 complex [12]. Consistent with previous reports emphasizing the significance of defined post-translational EZH2 modifications for its chromatin-, PRC2- or/and methyltransferase-independent functions [43,60,77,78], robust EZH2-NFATc1 complex formation requires post-translational EZH2 phosphorylation on the serine 21 residue [12]. Importantly, those post-translational EZH2 modifications are installed by kinases and other enzymes (e.g., AKT [43,60] or GSK3β [78]), thus emphasizing the significance of those hierarchical signaling cues for driving non-canonical EZH2 activities. Consequently, in addition to the elucidation of the molecular prerequisites predicting anti-tumorigenic consequences of EZH2 blockade, the distinction between canonical and non-canonical EZH2 activities contributing to tumor progression is relevant for the selection of the most efficient pharmacological strategies aiding at EZH2. Tazemetostat, although clinically approved for and potent in selected cancer entities predominantly characterized by canonical EZH2 activities, specifically blocks EZH2 histone methyltransferase activity, but does not interfere with EZH2 expression [79]. The awareness of the tumor-biological implications of non-canonical EZH2 functions in cancer progression resulted in the development of next-generation EZH2 inhibitors, which degrade the EZH2 protein [80], and hence are more likely to also target non-canonical EZH2 activities. In light of these developments, the thorough elucidation of the molecular subtypes and the context-defining cues determining canonical or non-canonical oncogenic EZH2 activity in PDAC and other malignancies might pave the way for the stratified and efficient implementation of EZH2-targeting in cancer treatment.

## Data Availability

Publicly available datasets were analyzed in this study. The accession number for RNA-seq data files is GSE197006. Furthermore, publicly available shRNA-mediated EZH2 knockdown RNA-seq data was used and can be found here: GSE153491.

## Supplementary Materials

The following supporting information can be downloaded at: https://www.mdpi.com/article/10.3390/cancers14143451/s1, Supplementary Figure S1: Quantification of (immuno-) histological stainings depicted in Figure 1G. Supplementary Figure S2: TP53-status-dependent EZH2 target gene regulation. Supplementary Figure S3: EZH2 depletion induces apoptosis-related protein expression in p53wt PDAC, but not in cells bearing mutations in the *Trp53* gene. Supplementary Figure S4: EZH2-dependent target gene regulation differs in p53wt and p53mut PDAC. Supplementary Figure S5: Un-cut original Western blot images. Supplementary Figure legends: Contains Figure legends for Supplementary Figures. Supplementary Table S1: sgRNA sequences and validation primers for CRISPR/Cas9-mediated EZH2 knockout. Supplementary Table S2: siRNA sequences. Supplementary Table S3: Primer for qRT-PCR. Supplementary Table S4: Antibodies used for western blotting. Supplementary Table S5: Antibodies used for immunohistochemistry and immunofluorescence. Supplementary Table S6: Antibodies used for ChIP experiments. Supplementary Table S7: Primer for qRT-PCR following ChIP experiments. Supplementary Table S8: Favorable prognosis genes depicted in Figure 2C,D. Supplementary Table S9: Genes being significantly upregulated in p53wt but not in p53mut cells upon EZH2 knockdown.
