# Supplementary material for: TP53-Status-Dependent Oncogenic EZH2 Activity in Pancreatic Cancer
Source: Cancers (Basel). 2022 Jul 15;14(14):3451. doi: 10.3390/cancers14143451 (PMC9320674; doi:10.3390/cancers14143451)
Supplement: Supplementary file 1 [file cancers-14-03451-s001.zip › cancers-1788033-supplementary.pdf]

cancers MDPI

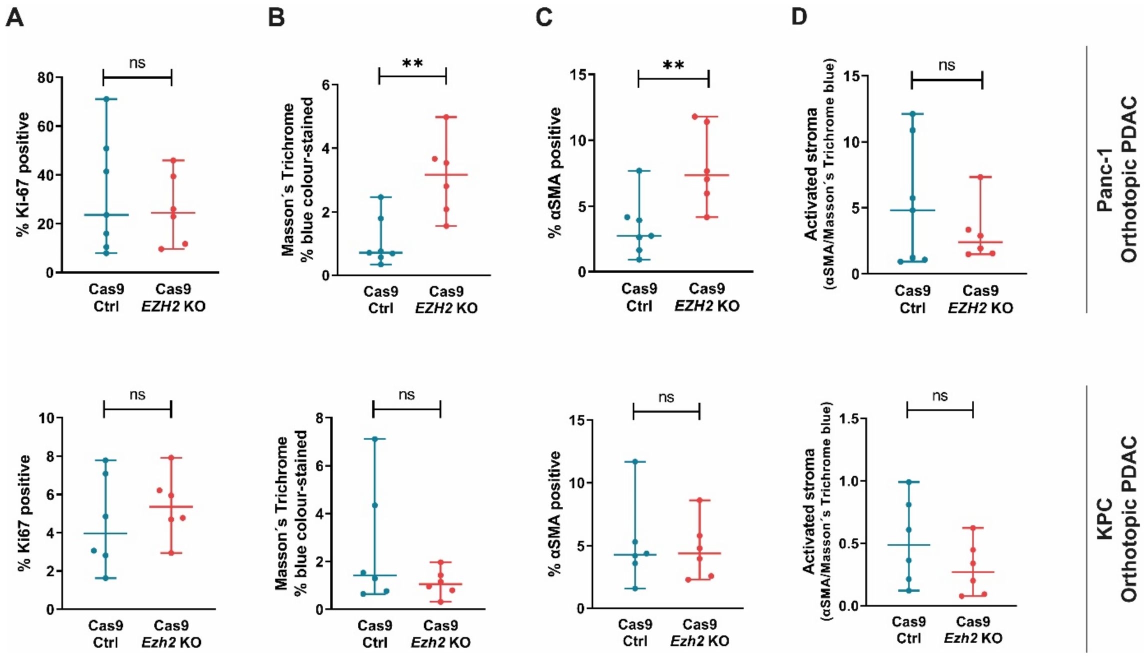

Supplementary Figure S1. Quantification of (immuno-) histological stainings depicted in Figure 1G. **A-C** Quantification of Ki-67 positive staining (**A**), Masson's Trichrome staining (**B**), and αSMA expression (**C**) of PDAC from orthotopically transplanted Panc1 cells (upper panel) and KrasG12D;Trp53R172H/+ (KPC) cells (lower panel). **D** Calculation of the activated stroma index by dividing the αSMA positive area by Masson´s Trichrome blue area (collagen). For Masson's Trichrome and αSMA staining percentage of positively stained area of ten representative images of each tumor and for Ki-67 staining percentage of Ki-67 positive cells of ten representative images were measured using ImageJ Fiji. Each dot represents one mouse. Values represent mean ± SD. Significance was determined by two-tailed unpaired Student´s t test; **, $p \leq 0.01$; ns, non-significant.

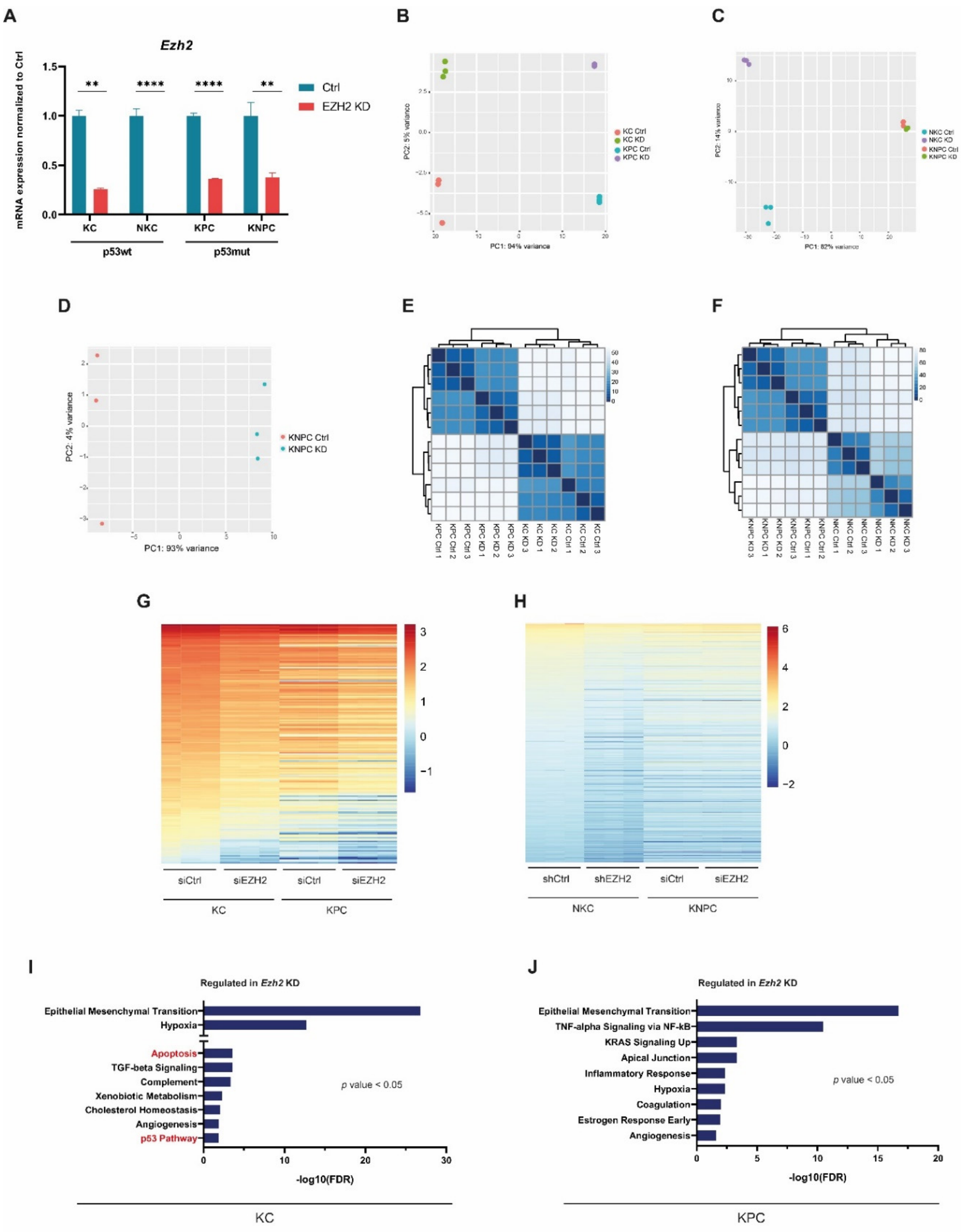

Supplementary Figure S2. *TP53*-status dependent EZH2 target gene regulation. **A** qRT-PCR analysis in the indicated p53wt and p53mut PDAC cells confirming knockdown of EZH2 (n = 3). Values represent mean ± SD. Significance was determined by Student´s t test; **, $p \leq 0.01$; ****, $p \leq 0.0001$. **B-D** Principal component analysis (PCA) upon RNA-sequencing of KC and KPC cells (**B**) and NKC and KNPC cells (**C, D**) after EZH2 knockdown displaying distinct clusters of triplicates. **E, F** Sample-to-sample distances upon RNA-seq analysis of KC and KPC cells (**E**) and NKC and KNPC cells (**F**) upon EZH2 knockdown. **G, H** Heatmap illustrating genes being significantly downregulated (FPKM >0.01, log2FC <-0.5, q <0.05) upon knockdown of EZH2 in the indicated p53wt cells (KC: 188 genes, NKC: 964 genes) and its consequences on the expression of these genes in the respective p53mut cells. **I, J** Gene ontology (GO) analysis to reveal significantly upregulated pathways upon EZH2 depletion in the indicated PDAC cells (p <0.05).

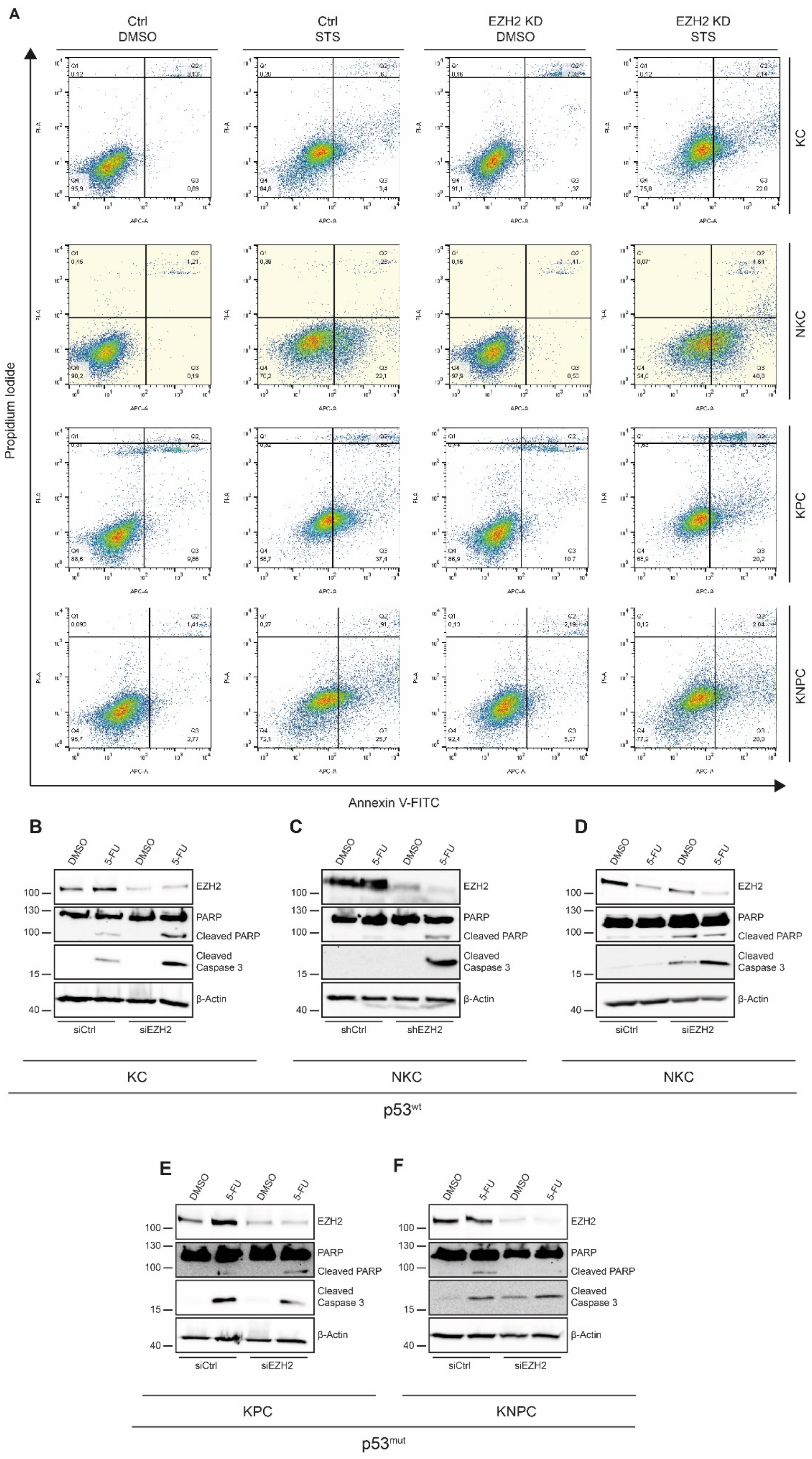

Supplementary Figure S3. depletion induces apoptosis-related protein expression in p53wt PDAC, but not in cells bearing mutations in the *Trp53* gene. **A** Knockdown of EZH2 in PDAC cells with

p53wt (KC, NKC) and p53mut (KPC, KNPC) expression and simultaneous treatment with staurosporine (STS). Representative flow cytometry results with the respective gating strategy upon Annexin-V/propidium iodide staining for each cell line referring to Figure 3. **B-F** Western blot analysis of apoptosis-related proteins upon knockdown of EZH2 and simultaneous treatment with 5-FU in the indicated p53wt and p53mut PDAC cells.

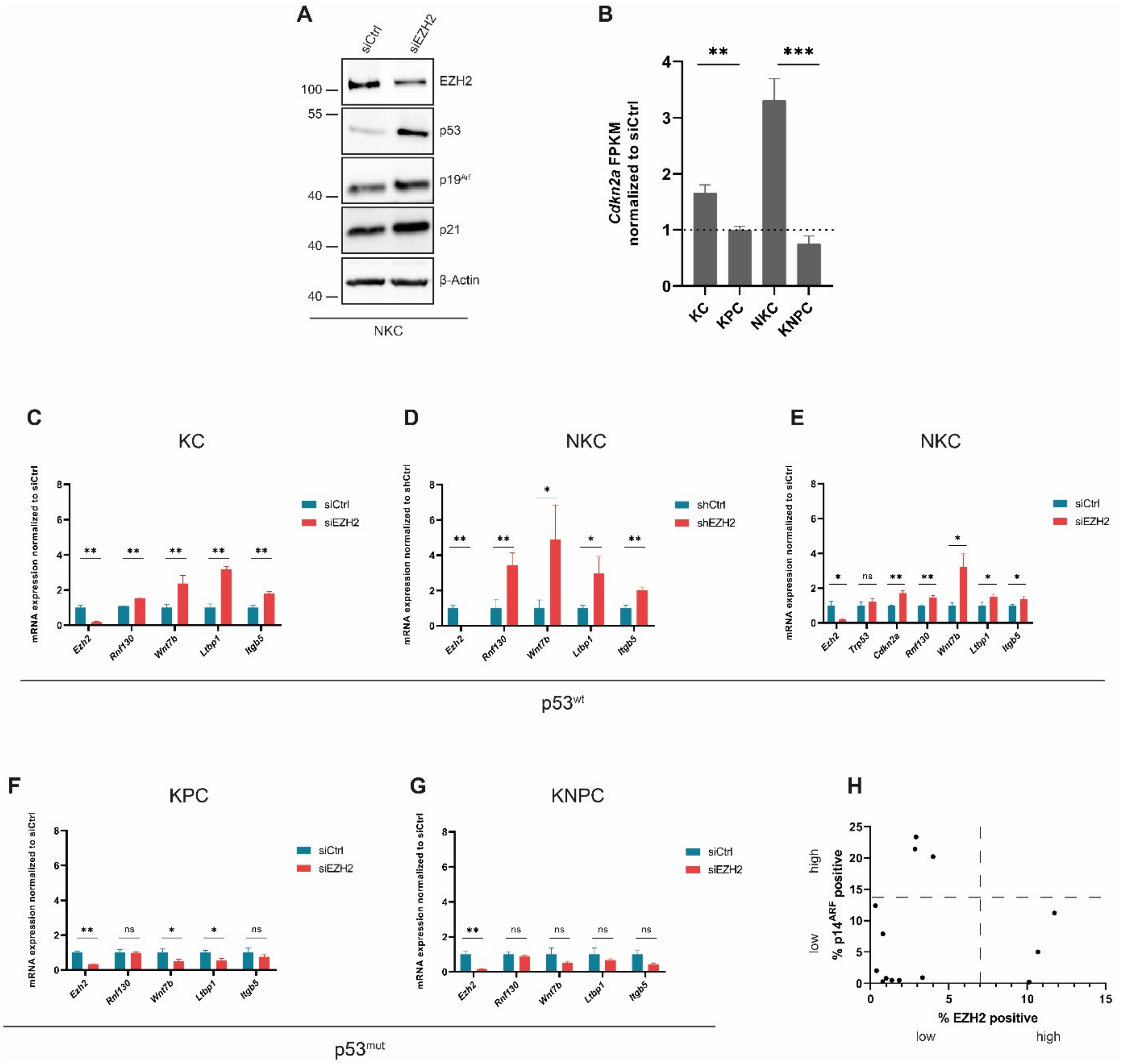

Supplementary Figure S4. EZH2-dependent target gene regulation differs in p53wt and p53mut PDAC. **A** Knockdown of EZH2 in p53wt NKC cells using siRNA following western blot analysis to investigate the consequences of EZH2 KD on the indicated proteins. **B** Normalized FPKM values of Cdkn2a expression after knockdown of EZH2 as revealed by RNA-seq in the indicated cell lines. Values represent mean ± SD. Significance was determined by Student´s t test; **, p ≤ 0.01; ***, p ≤ 0.001. **C-G** QRT-PCR analysis in the indicated PDAC cells upon knockdown of EZH2 validating the upregulation of these genes in p53wt cells (n = 3). Values represent mean ± SD. Significance was determined by Student´s t test; *, p ≤ 0.05; **, p ≤ 0.01; ns, non-significant. **H** Table displaying number of patients in the indicated EZH2low/high and p14ARF low/high groups based on IHC staining.

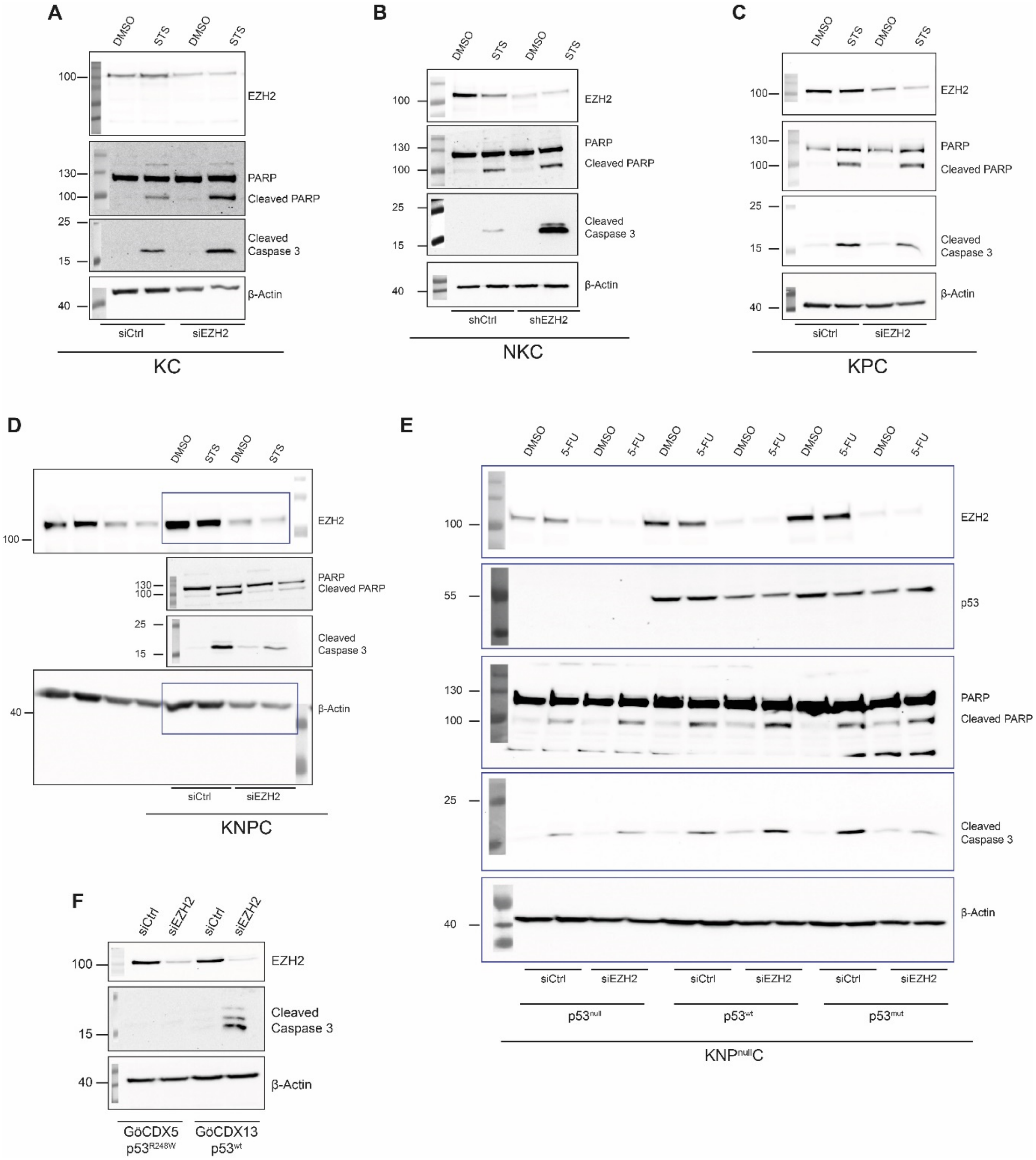
A
DMSO
STS
DMSO
STS
100
EZH2
130
100
PARP
Cleaved PARP
25
15
Cleaved Caspase 3
40
β-Actin
siCtrl
siEZH2
KC
B
DMSO
STS
DMSO
STS
100
EZH2
130
100
PARP
Cleaved PARP
25
15
Cleaved Caspase 3
40
β-Actin
shCtrl
shEZH2
NKC
C
DMSO
STS
DMSO
STS
100
EZH2
130
100
PARP
Cleaved PARP
25
15
Cleaved Caspase 3
40
β-Actin
siCtrl
siEZH2
KPC
D
DMSO
STS
DMSO
STS
100
EZH2
130
100
PARP
Cleaved PARP
25
15
Cleaved Caspase 3
40
β-Actin
siCtrl
siEZH2
KNPC
E
DMSO
5-FU
DMSO
5-FU
DMSO
5-FU
DMSO
5-FU
DMSO
5-FU
DMSO
5-FU
100
EZH2
55
p53
130
100
PARP
Cleaved PARP
25
Cleaved Caspase 3
40
β-Actin
siCtrl
siEZH2
siCtrl
siEZH2
siCtrl
siEZH2
$p53^{null}$
$p53^{wt}$
$p53^{mut}$
$KNP^{null}C$
F
siCtrl
siEZH2
siCtrl
siEZH2
100
EZH2
15
Cleaved Caspase 3
40
β-Actin
GöCDX5
$p53^{R248W}$
GöCDX13
$p53^{wt}$

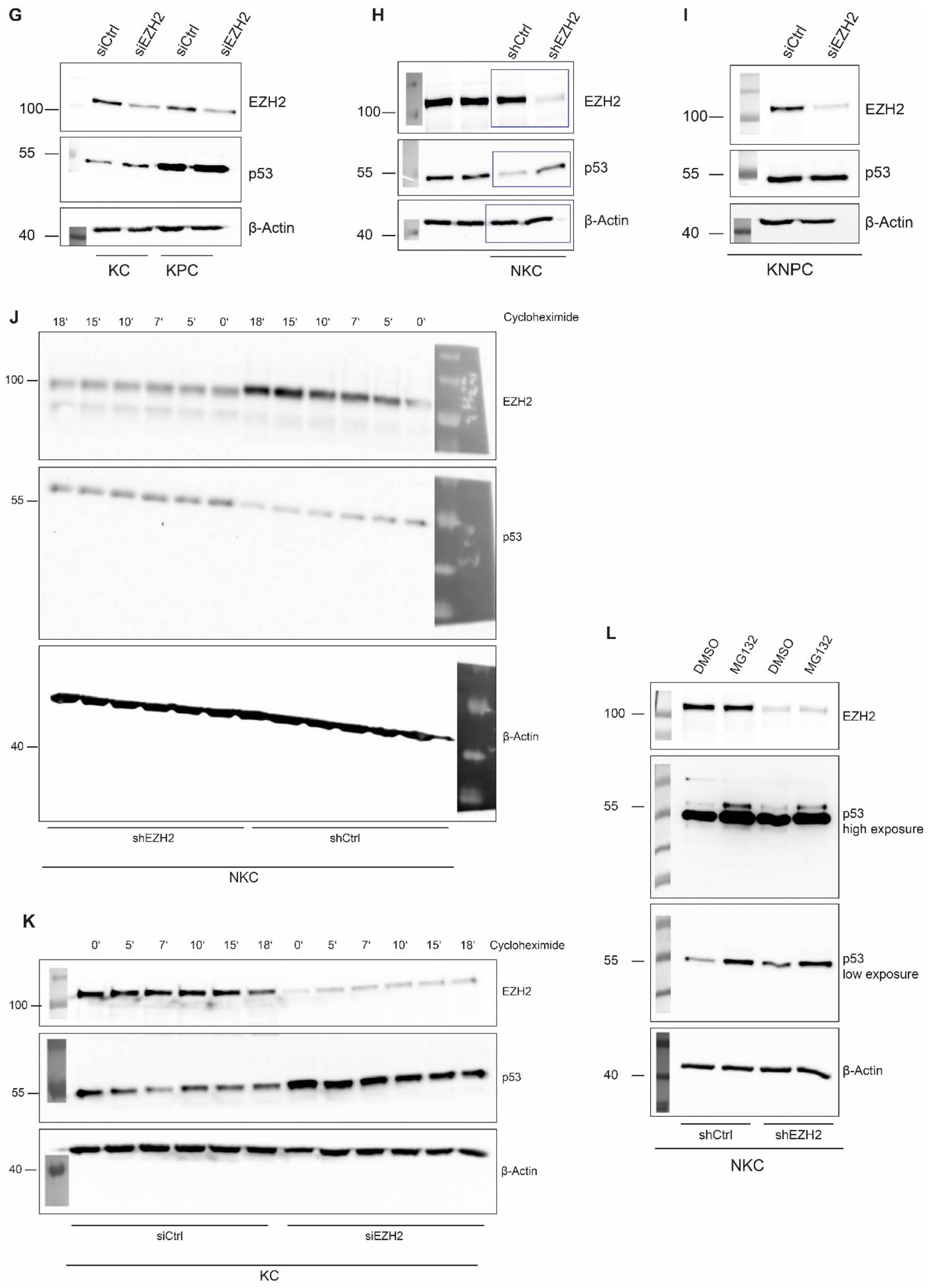
G
siCtrl
siEZH2
siCtrl
siEZH2
100—
EZH2
55 —
p53
40 —
β-Actin
KC
KPC
H
shCtrl
shEZH2
100—
EZH2
55 —
p53
40 —
β-Actin
NKC
I
siCtrl
siEZH2
100—
EZH2
55 —
p53
40 —
β-Actin
KNPC
J
18' 15' 10' 7' 5' 0' 18' 15' 10' 7' 5' 0'
Cycloheximide
100 —
EZH2
55 —
p53
40 —
β-Actin
shEZH2
shCtrl
NKC
K
0' 5' 7' 10' 15' 18' 0' 5' 7' 10' 15' 18'
Cycloheximide
100 —
EZH2
55 —
p53
40 —
β-Actin
siCtrl
siEZH2
KC
L
DMSO
MG132
DMSO
MG132
100 —
EZH2
55 —
p53
high exposure
55 —
p53
low exposure
40 —
β-Actin
shCtrl
shEZH2
NKC

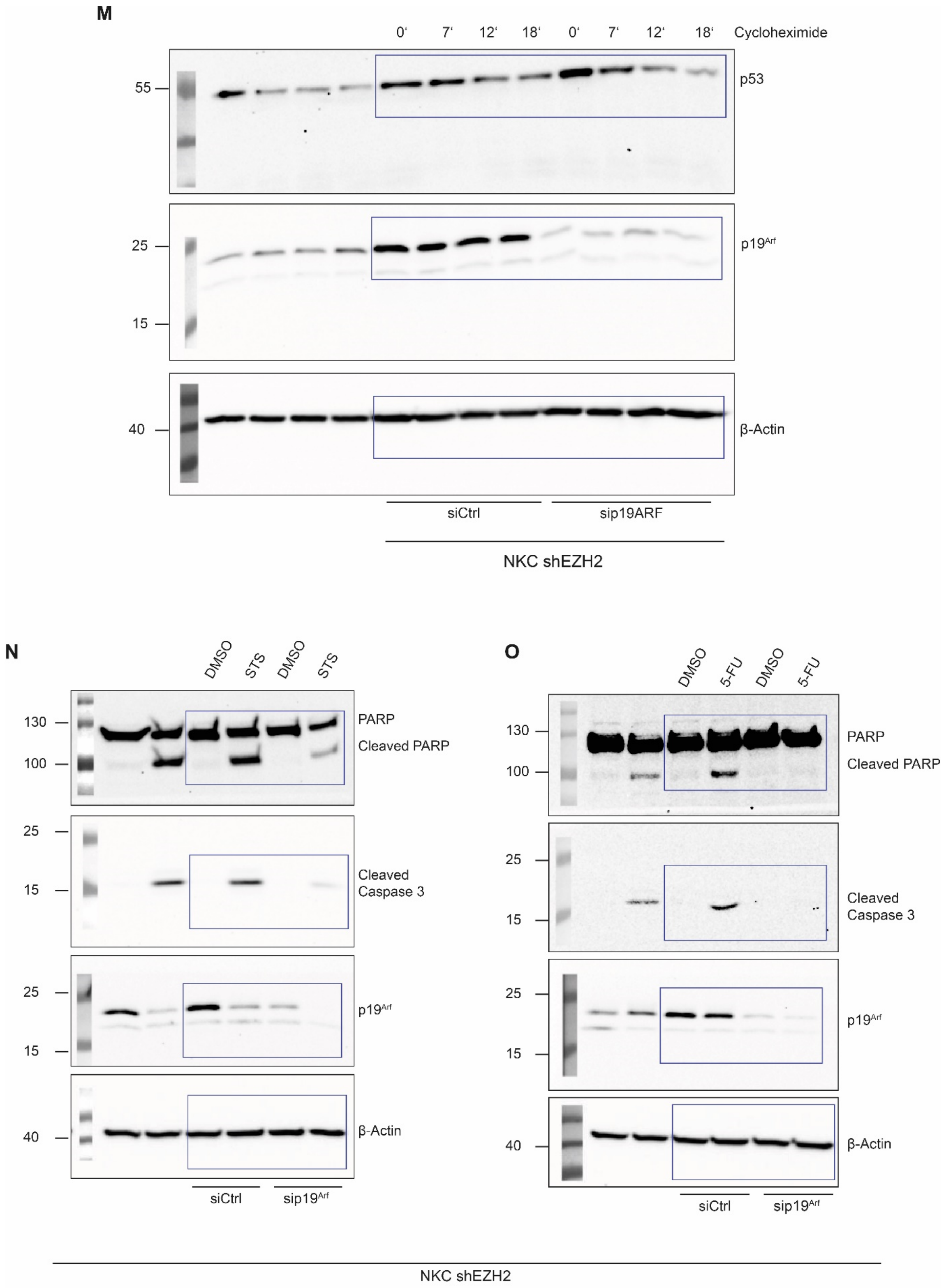
M
0' 7' 12' 18' 0' 7' 12' 18' Cycloheximide
55
p53
25
15
p19Arf
40
β-Actin
siCtrl
sip19ARF
NKC shEZH2
N
DMSO STS DMSO STS
130
100
PARP
Cleaved PARP
25
15
Cleaved Caspase 3
25
15
p19Arf
40
β-Actin
siCtrl
sip19Arf
O
DMSO 5-FU DMSO 5-FU
130
100
PARP
Cleaved PARP
25
15
Cleaved Caspase 3
25
15
p19Arf
40
β-Actin
siCtrl
sip19Arf
NKC shEZH2

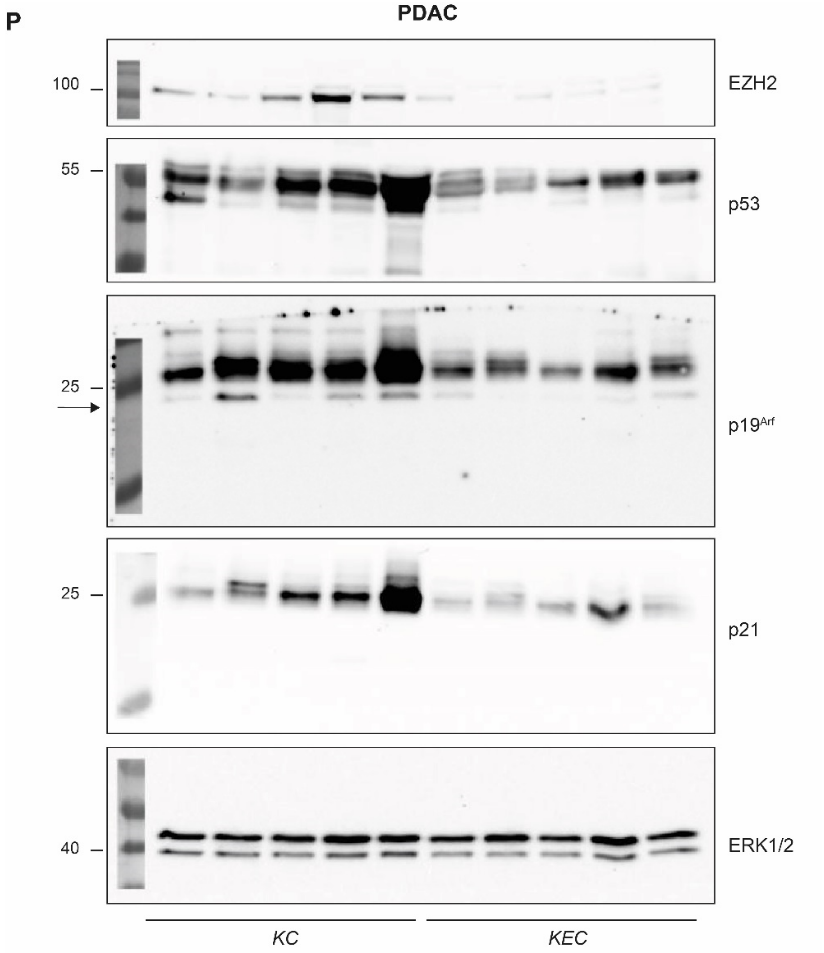
P
PDAC
100
EZH2
55
p53
25
p19Arf
25
p21
40
ERK1/2
KC
KEC

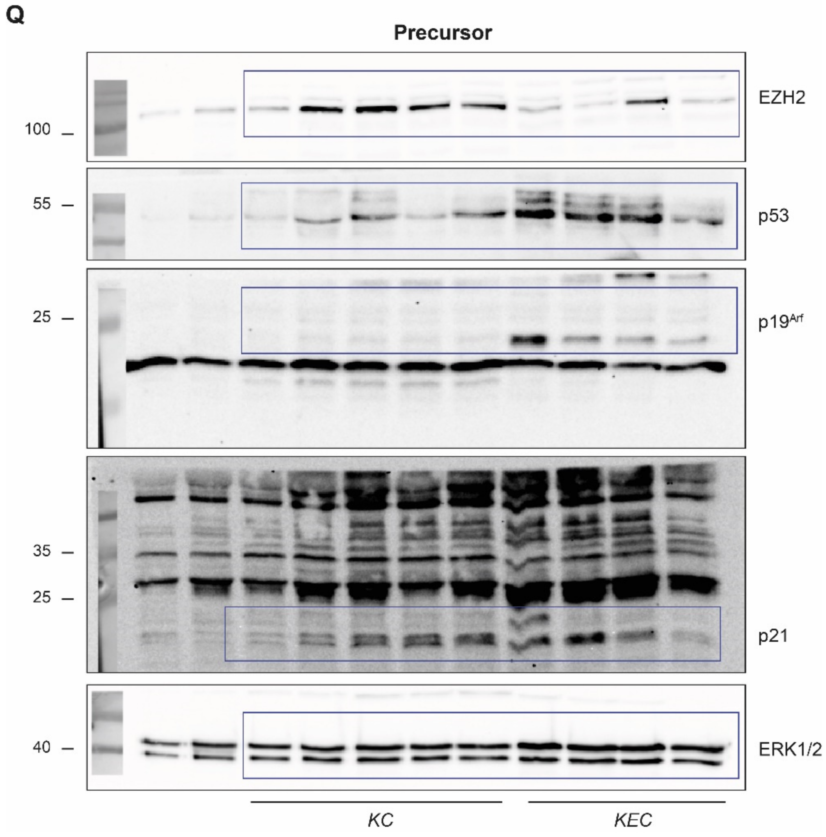
Q
Precursor
EZH2
100
55
p53
25
p19Arf
35
25
p21
40
ERK1/2
KC
KEC

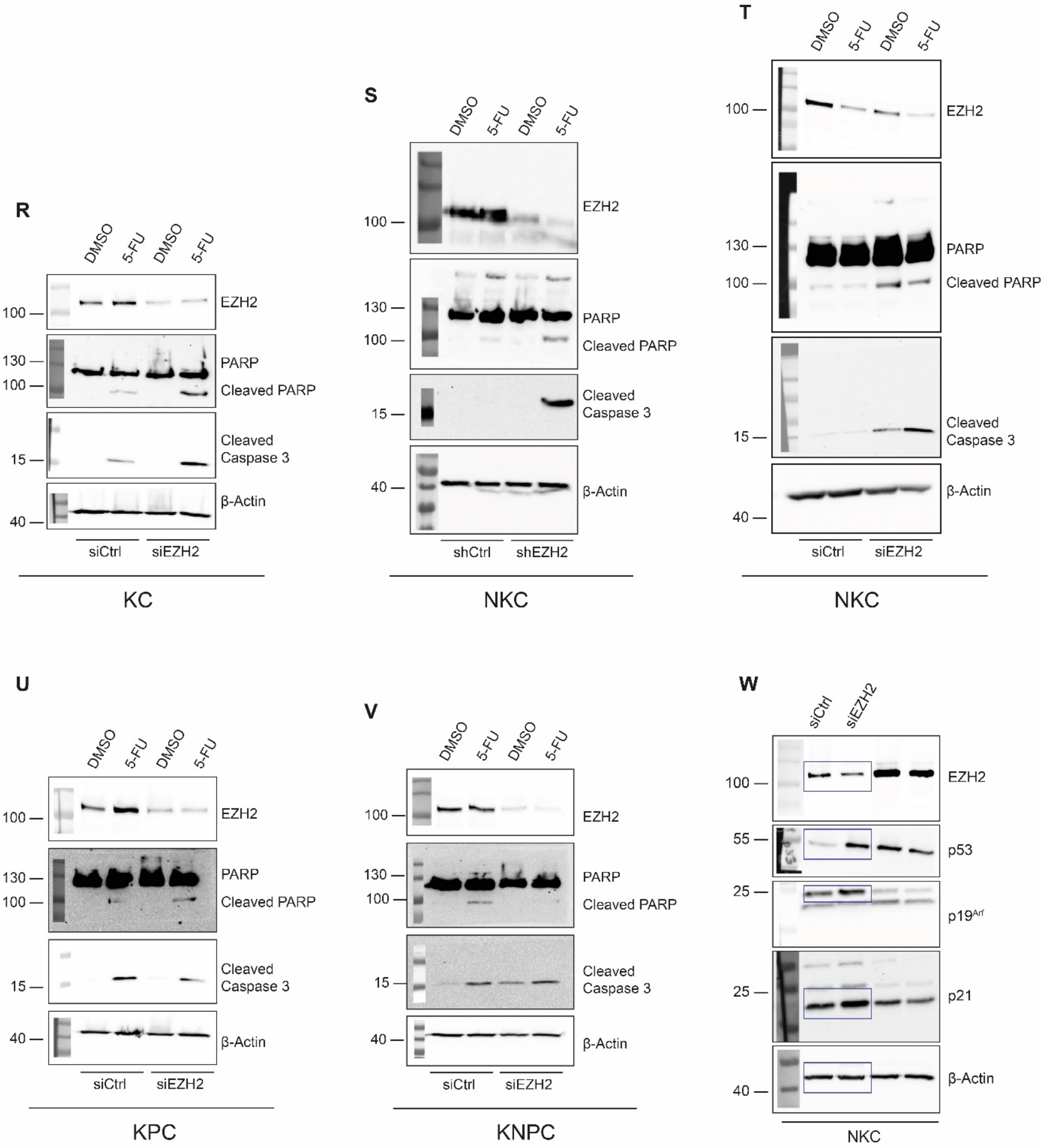

Supplementary Figure S5. Un-cut original Western Blot images. Original western blot images from those western blots shown throughout the manuscript. We used the PageRule Prestained Protein Ladder, 10 bis 180 kDa from Thermo Scientific (26616) having bands at 180, 130, 100, 70, 55, 40, 35, 25, 15, and 10 kDa. We did not label all bands of the molecular weight marker but only the most relevant. However, the other molecular weight sizes can be identified accordingly. For protein visualization, we used the Western Lightning Plus (NEL103E001EA) or Ultra (NEL111001EA) chemiluminescent substrate from PerkinElmer. However, the marker cannot be visualized with the chemiluminescent solution and was imaged separately. Subsequently, we added the marker from the raw colorimetric image onto the enhanced chemiluminescent image to compare the molecular weight of the protein of interest with the standard. Please note, that we pre-cut the membranes before antibody incubation around the expected molecular weight of the protein of interest in order to stain different proteins of distinct molecular weight on one membrane. Blue boxes indicate excerpt of protein lanes depicted in the respective Figures. **A** Un-cut western blot images referring to Fig. 3C. **B** Un-cut western blot images referring to Fig. 3E. **C** Un-cut western blot images referring to Fig. 3G. **D** Un-cut western blot images referring to Fig. 3I. **E** Un-cut western blot images referring to Fig. 3K. **F** Un-cut western blot images referring to Fig. 3L. **G** Un-cut western blot images referring to Fig. 4A

and 4C. **H** Un-cut western blot images referring to Fig. 4B. **I** Un-cut western blot images referring to Fig. 4D. **J** Un-cut western blot images referring to Fig. 4F. **K** Un-cut western blot images referring to Fig. 4G. **L** Un-cut western blot images referring to Fig. 4H. **M** Un-cut western blot images referring to Fig. 5E. **N** Un-cut western blot images referring to Fig. 5F. **O** Un-cut western blot images referring to Fig. 5G. **P** Un-cut western blot images referring to Fig. 6B. **Q** Un-cut western blot images referring to Fig. 6H. **R** Un-cut western blot images referring to Supplementary Fig. 3B. **S** Un-cut western blot images referring to Supplementary Fig. 3C. **T** Un-cut western blot images referring to Supplementary Fig. 3D. **U** Un-cut western blot images referring to Supplementary Fig. 3E. **V** Un-cut western blot images referring to Supplementary Fig. 3F. **W** Un-cut western blot images referring to Supplementary Fig. 4A.

Supplementary tables

Supplementary Table S1. sgRNA sequences and validation primers for CRISPR/Cas9-mediated *EZH2* knockout.

	Murine *Ezh2*	Human *EZH2*
**sgRNA sequence**	GTGGTGGATGCAACCCGAAA	GTGGTGGATGCAACCCGCAA
**Forward primers for knockout validation**	CCTGTGTAAGTGGGTGTGCT	TGCCTATTCGTGATGTTTGGAAG
**Reverse primers for knockout validation**	GTTTGCTGTCACTTGGCTGG	TGTCAACAGCAGGGTGAGAAA

Supplementary Table S2. siRNA sequences.

	Sense	Antisense
**siEZH2**	GGAUACAGCCUGUGCACAUTT	AUGUGCACAGGCUGUAUCCTC
**sip19^{Arf}**	GGCUAGAGAGGAUCUUGAGTT	CUCAAGAUCCUCUCUAGCCTC

Supplementary Table S3. Primer for qRT-PCR.

Target	Direction	Sequence
Cdkn2a	forward	CGCAGGTTCTTGGTCACTGT
Cdkn2a	reverse	TGTTCACGAAAGCCAGAGCG
Ezh2	forward	CAACCCGAAAGGGCAACAAA
Ezh2	reverse	ACC AGT CTG GAT AGC CCT CT
Itgb5	forward	GAAGTGCCACCTCGTGTGAA
Itgb5	reverse	GGACCGTGGATTGCCAAAGT
Ltbp1	forward	GGTCGCATCAAGGTGGTCTTT
Ltbp1	reverse	GTGGTGGTATTCCCCTTCTGG
Rnf130	forward	CTGCCCATCCACGGAGTTG
Rnf130	reverse	CAAGGCGATCCACTGTTTGA
Rplp0	forward	TGGGCAAGAACACCATGATG
Rplp0	reverse	AGTTTCTCCAGAGCTGGGTTGT
Trp53	forward	AGGTGTGCGTAGCACC
Trp53	reverse	CCCCACAACACCAGTG
Wnt7b	forward	CTTCACCTATGCCATCACGG
Wnt7b	reverse	TGGTTGTAGTAGCCTTGCTTCT

Supplementary Table S4. Antibodies used for western blotting.

Antibody	Company	Number	Dilution
Actin-HRP	Sigma	A3854	1:40000
Cleaved caspase 3	Cell Signaling	9661	1:500
Erk (1/2)	Cell Signaling	9102	1:1000
EZH2	Cell Signaling	5246	1:1000
p19^{Arf}	Abcam	Ab80	1:1000
p19^{Arf}	Santa Cruz	32748	1:250
p53	Cell Signaling	2524	1:1000
PARP	Cell Signaling	9542	1:1000
Anti-mouse (IgG) HRP	Cell Signaling	7076	1:6500
Anti-rabbit (IgG) HRP	Cell Signaling	7074	1:6500
Anti-rat (IgG) HRP	Santa Cruz	2006	1:5000

Supplementary Table S5. Antibodies used for immunohistochemistry and immunofluorescence.

Antibody	Company	Number	Dilution
EZH2 (mouse)	Cell Signaling	5246	1:100
EZH2 (human)	Leica Biosystems	NCL-L-EZH2	1:50
Ki-67	Thermo Fisher	RM9106	1:600
αSMA	Agilent Dako	M0851	1:100
p14	Cell Signaling	2407	1:400
$p19^{Arf}$	Santa Cruz	32748	1:100
Alexa Fluor 488 donkey anti-rat	Invitrogen	A21208	1:500

Supplementary Table S6. Antibodies used for ChIP experiments.

Antibody	Company	Number	Amount
EZH2	Diagenode	C15410039-classic	2 µg
H3K4me3	Cell Signaling	9751	2 µg
Rabbit IgG	Diagenode	C15410206	2 µg

Supplementary Table S7. Primer for qRT-PCR following ChIP experiments.

Target	Direction	Sequence
Cdkn2a TSS	forward	GACCGTGAAGTTCAGC
Cdkn2a TSS	reverse	GGGGTCGCTTCTTCGG

Supplementary Table S8. Favorable prognosis genes depicted in Figure 2C/D.

CAMTA2	*ABHD8*	*HCFC1*	*VPS54*	*SNAI3*	*GSE1*	*TBPL1*
TSPYL2	*SEMA6C*	*PPP1R10*	*MCM3AP*	*DOHH*	*LRRC29*	*ARIH2*
ANAPC2	*TMEM74B*	*SAFB*	*PMM1*	*RFX6*	*UAP1L1*	*RFX2*
DEF8	*GALT*	*KLHL36*	*ATP6V0A1*	*HERC1*	*KIF5A*	*SLC22A5*
USP20	*COG8*	*TMEM175*	*COQ10A*	*PRPSAP2*	*SLC27A3*	*DOPEY1*
MICAL1	*RAB11FIP3*	*ABCA5*	*RAB4B*	*UNC13B*	*DYNLL2*	*BAHCC1*
SOCS2	*EPOR*	*SSBP4*	*NEIL1*	*GNG7*	*PNISR*	*ASPHD1*
SLC43A2	*DENND4B*	*BRSK1*	*RBM4B*	*SNTA1*	*NCALD*	*MPDZ*
ARNT2	*NECAB3*	*BBS5*	*QTRT1*	*TACO1*	*INPP5B*	*MLLT6*
WDR37	*NAT9*	*TYK2*	*SPATC1L*	*NCDN*	*RAD51C*	*SMPD1*
INPP5K	*NFASC*	*CBX7*	*ZBTB46*	*HNRNPA0*	*TRMT10B*	*ULK3*
PITPNA	*PKD1*	*RNF166*	*CXXC4*	*ALKBH5*	*TAPT1*	*XAB2*
PPP1R3F	*ARMCX2*	*RBM14*	*IPO13*	*PSPH*	*TSPYL1*	*PPP6R2*
MAMDC4	*PNPLA6*	*ATP1B2*	*BTBD6*	*MAVS*	*POLR3H*	*MAP2K6*
ARMC5	*TMEM91*	*APH1B*	*WHRN*	*DVL2*	*NAP1L2*	*ANKRD13B*
RNF167	*B4GAT1*	*PLD6*	*PIP5K1C*	*CD81*	*INTS3*	*RBM5*
CCDC106	*IRGQ*	*PTPRS*	*IRF2BP1*	*GOLGA3*	*WDR83*	*RMND5B*
MUM1	*INCA1*	*ZFP3*	*ST3GAL2*	*FRS3*	*PFDN5*	*GAS8*
SFXN5	*RNASEH2C*	*GDF11*	*EFHC1*	*DIDO1*	*ANGEL1*	*PHKA2*
TLE2	*ACSF3*	*NCOA5*	*SDR39U1*	*IQCD*	*SYT5*	*GPX4*
CBX6	*GADD45G*	*NICN1*	*FOXA2*	*IGIP*	*NELL2*	*FKBP8*
KCND1	*CBX8*	*HDAC4*	*CCDC130*	*ADD1*	*REC8*	*RNPC3*
RBM10	*POMT1*	*KLHDC4*	*CERK*	*MAGEE1*	*HEXDC*	*ATP6AP1*
TBC1D13	*ARGLU1*	*ADGRL1*	*FN3K*	*HDAC3*	*SCG2*	*MC1R*
RAD51D	*PELP1*	*LIPE*	*SYNGR1*	*HIRA*	*STK40*	*MVB12B*
FXR2	*OAZ1*	*CCM2*	*FAM219A*	*FASTK*	*KCNH2*	*PDE3B*
VAMP2	*PPP1R12C*	*MBLAC1*	*NUDT18*	*TBC1D17*	*CALM1*	*SPECC1L*
KLHL22	*ZBTB40*	*DNMT3A*	*DCLK2*	*PRKAR2B*	*NEK9*	*MZF1*
RAB6B	*SPATA7*	*GRK6*	*RCAN2*	*SOWAHA*	*B4GALT6*	*DCAF15*
EVL	*MED9*	*SALL2*	*NPR1*	*ENPP5*	*TEX264*	*MIS12*
TMEM203	*KCTD17*	*RGS11*	*SLC25A44*	*NXF1*	*TMEM150C*	*TSC1*
PRAF2	*GPR162*	*MKS1*	*ID4*	*RAB17*	*CLEC3B*	*PHKG2*
CYB561D1	*MCF2L*	*UNC119B*	*TRIM39*	*RANBP3*	*JMJD6*	*AKAP8L*
ZBTB48	*SNAPC4*	*LUC7L*	*PIGT*	*RFXAP*	*TUBGCP6*	*PPP1R21*
ELMOD3	*SNRNP70*	*RAPGEF4*	*THEM4*	*MRPL38*	*ITGAE*	*CERS4*
PHLDB3	*TTC13*	*TBL1X*	*CHPF2*	*DEXI*	*ATP6V0E2*	*CUL9*
SLC25A11	*CCDC92*	*SPPL2B*	*TMEM240*	*ACCS*	*SFI1*	*FBXO46*
VASH1	*MTHFR*	*RGS9*	*CCND2*	*HAP1*	*SMDT1*	*TECPR1*

LRSAM1	*LPCAT1*	*MRPL53*	*SAFB2*	*POMGNT1*	*DXO*	*LENG1*
CBFA2T2	*PKIG*	*SCN1B*	*ZBTB4*	*NAA60*	*GPANK1*	*SRR*
FAM53B	*USF2*	*RWDD2A*	*CLDN15*	*CACNA1A*	*DNAJC18*	*OTUD5*
BRF1	*RNF146*	*TOP3A*	*EID2B*	*RECQL5*	*IGHMBP2*	*FZD8*
NUAK2	*MAP1LC3A*	*GCH1*	*MAGEH1*	*IKZF4*	*GNAO1*	*EFNB3*
TBKBP1	*STRADA*	*RBM6*	*CXXC1*	*SIN3B*	*CEP164*	*WRAP53*
DLG4	*TRIM3*	*CCDC159*	*ENGASE*	*SH2B1*	*GFOD2*	*MRPL57*
ATOH8	*CCDC57*	*DTX3*	*ELP5*	*CRY2*	*FXYD6*	*CHD3*
SGSM2	*MCOLN1*	*PCGF3*	*FBXO44*	*EMC10*	*NEU3*	*CNPY2*
NCAM1	*PRR3*	*RABL6*	*PBXIP1*	*RANGRF*	*ZDHHC1*	*FIZ1*
CEP250	*DCUN1D2*	*TERF2IP*	*MAP1S*	*UCKL1*	*GBA2*	*KATNB1*
B3GNT8	*SCAMP5*	*NINL*	*CAMKK1*	*SIRT3*	*SLC30A4*	*STARD3NL*
SLC2A8	*MADD*	*SLC16A13*	*NT5C3B*	*FLCN*	*EPC1*	*SHC2*
TMEM86B	*PTCH2*	*APBA3*	*ASPSCR1*	*SLC29A4*	*PLEKHB1*	*SDSL*
NAIF1	*VPS16*	*MAP3K12*	*CIRBP*	*TMOD2*	*MEGF8*	*KDM2B*
SEC61A2	*PCED1A*	*PEBP1*	*CDK10*	*ALDH9A1*	*LUC7L3*	*PAF1*
CCDC28B	*LLGL2*	*EXOC3*	*FAM120B*	*AP2A2*	*ZZEF1*	*R3HCC1*
PHF10	*SAT2*	*ASB1*	*MIF4GD*	*ARFGAP1*	*ELMO2*	*VPS18*
CLSTN3	*MOCS1*	*ATP8B2*	*WDR19*	*ATP5D*	*RGL2*	*TMEM59L*
ATP6V0D1	*ANKRD39*	*ING5*	*METTL3*	*ELMO1*	*SLC25A27*	*TPGS1*
BCAS3	*ATP1A3*	*MYBBP1A*	*KRBA1*	*ACBD4*	*ANKRD54*	*IRX2*
PI4KB	*SENP3*	*STK11IP*	*RAI2*	*TATDN2*	*GPR137*	*TSPAN33*
KCTD2	*RNF216*	*STAT5B*	*MINK1*	*FBXW4*	*SUOX*	*FKRP*
CHST12	*MTG2*	*LIN37*	*MAGED2*	*DCXR*	*LRRC56*	*NPM2*
GIGYF1	*CTNS*	*IZUMO4*	*SLC25A45*	*DCAF8*	*GPATCH3*	*SREBF1*
MXD4	*CTC1*	*TSPAN7*	*FAAP20*	*PLCG1*	*RABEP1*	*KCTD13*
SLC26A11	*IFFO1*	*DPY19L3*	*SLC25A29*	*ASCL2*	*PLD3*	*IQSEC1*
SLC16A11	*TNRC6C*	*PGPEP1*	*SLC38A10*	*TFEB*	*MED22*	*HELQ*
SOBP	*UNK*	*GTPBP6*	*PACS2*	*TAOK2*	*PRKACA*	*KLHDC3*
VPS53	*PPP3CB*	*TNS2*	*CENPT*	*FYN*	*POLR3F*	*TRAPPC12*
ZMAT1	*GKAP1*	*ARHGEF11*	*TANGO2*	*DIRAS1*	*CLPP*	*PTOV1*
ABHD17A	*UPF3A*	*TMEM220*	*SMARCA1*	*MAN1C1*	*DPH7*	*HDAC10*
MBD3	*SLC23A2*	*SLC22A17*	*METTL16*	*FAM193A*	*PAPSS2*	*SPHK2*
GPRASP1	*NMNAT3*	*KDM8*	*SGSH*	*EGLN2*	*AXIN2*	*USP27X*
DPH1	*ARMCX1*	*ITGA7*	*PPP1R9B*	*RFNG*	*AKT1S1*	*TDRP*
CRTC1	*PGS1*	*TAF1C*	*TBCB*	*FAM222A*	*POU6F1*	*ZFAND2B*
UBOX5	*FBXL16*	*ZBTB49*	*SEPHS1*	*INTS1*	*KBTBD7*	*FAM83F*
APBB1	*LRRC75A*	*ZSWIM1*	*TFPT*	*GGA1*	*SSTR2*	*RASIP1*
FLYWCH2	*GPS2*	*ZSWIM7*	*TMEM8B*	*NCKAP5L*	*PKNOX1*	*LYRM9*
EXOG	*AHI1*	*CFD*	*NPDC1*	*MRPL34*	*IMMP1L*	*GTF3C1*
ARID3A	*RAB11B*	*SLC25A14*	*ATP6V0B*	*CCDC96*	*KLC1*	*ATOX1*
TMEM104	*CYB5D2*	*LRRC14*	*PIN1*	*SORBS3*	*PGBD1*	*AGFG2*
ARAP1	*CAPN10*	*CTDNEP1*	*KCTD7*	*LSM10*	*APBB3*	*SGK3*
SAP30L	*PMPCA*	*FAM117A*	*ERCC2*	*NAAA*	*WBP1*	*CISH*
ENPP2	*RUFY3*	*INPP5E*	*MON1A*	*WNT4*	*IP6K1*	*NDUFA3*
VPS37D	*UNC119*	*FBXO9*	*WDR81*	*P3H3*	*TRMU*	*PABPN1*
CDIP1	*IQCC*	*CPT1C*	*ABCB8*	*SPG7*	*ACADVL*	*EFCC1*
TRAF1	*DPP7*	*DUSP28*	*TRIM25*	*RFX1*	*TMEM198*	*ROGDI*
MARK1	*TSPYL4*	*UQCR11*	*CSAD*	*PDZRN3*	*NAGLU*	*TIMM13*
PNPLA7	*ORAI2*	*CYB561D2*	*ACADSB*	*DISP1*	*PPP1R26*	*WIPI2*
GPR173	*U2AF1L4*	*YIF1B*	*MAP6*	*SMIM4*	*ZDHHC14*	*STXBP1*
NEURL4	*TXLNA*	*NISCH*	*RGAG4*	*STIM2*	*UBXN6*	*PALM*
TRAF3	*NRIP2*	*RNASEK*	*AGER*	*RSAD1*	*MAST3*	*ATG4D*
EBF4	*LRWD1*	*TOM1L2*	*SLC25A4*	*CPLX1*	*RXRA*	*CHGB*
CASKIN2	*FBXO31*	*TRO*	*TIMM22*	*TSC2*	*PIAS4*	*CHMP6*
GABBR1	*DMPK*	*ZCWPW1*	*MTERF4*	*MTG1*	*ATG16L2*	*SMPD2*
TSEN54	*FAM69B*	*ACACB*	*LMBR1L*	*MTMR4*	*SCAP*	
CD99L2	*MTA1*	*KLHDC1*	*ZSCAN2*	*IFT88*	*MST1*	
HSD17B14	*GABARAP*	*ZFP57*	*CALY*	*CLCN5*	*TBXA2R*	
PDZD4	*CSNK1D*	*ANKRD16*	*KAT2A*	*RPAIN*	*GRIK5*	

Supplementary Table S9. Genes being significantly upregulated in p53wt but not in p53mut cells upon EZH2 knockdown.

Anxa6	*Dtna*	*Igf2bp3*	*Maged2*	*Ppbp*	*Slfn2*	*Tpm2*
Camk2b	*Efemp2*	*Igf2r*	*Map1lc3a*	*Rdh10*	*Spon2*	*Trp53inp2*
Cdkn2a	*Fhdc1*	*Itgb5*	*Masp1*	*Rnf130*	*Sprr1a*	*Tuft1*
Ddah2	*Foxg1*	*Ltbp1*	*Neat1*	*Serpinb6b*	*Tbc1d16*	*Wnt7b*
Dnaaf9	*Fzd6*	*Ltbp3*	*Palld*	*Slc44a2*	*Tcf24*	*Zfpm2*
